# Low RNA disruption during neoadjuvant chemotherapy predicts pathologic complete response absence in patients with breast cancer

**DOI:** 10.1093/jncics/pkad107

**Published:** 2023-12-19

**Authors:** Marina Elena Cazzaniga, Foluso Ademuyiwa, Thierry Petit, Joke Tio, Daniele Generali, Eva M Ciruelos, Nadia Califaretti, Brigitte Poirier, Antonio Ardizzoia, Arnd Hoenig, Benno Lex, Marie-Ange Mouret-Reynier, Dagmar Giesecke, Nicolas Isambert, Ricardo Masetti, Lacey Pitre, Denise Wrobel, Paule Augereau, Manuela Milani, Sara Rask, Christine Solbach, Laura Pritzker, Sanaa Noubir, Amadeo Parissenti, Maureen E Trudeau

**Affiliations:** Phase 1 Research Unit, IRCCS San Gerardo dei Tintori, Monza, MB, Italy; School of Medicine and Surgery, Milano Bicocca University, Monza, MB, Italy; Division of Medical Oncology, Washington University, Saint Louis, MO, USA; Institut Cancérologie Strasbourg Europe, Strasbourg, France; Department of Gynecology and Obstetrics, Universitätsklinikum Münster, Münster, Germany; Breast Cancer Unit, ASST of Cremona and Department of Life Sciences, University of Trieste, Trieste, Italy; Medical Oncology Department, Breast Cancer Unit, University Hospital, 12 de Octubre, Madrid, Spain; Grand River Regional Cancer Centre, Kitchener, ON, Canada; CHU de Québec-Université Laval, Québec City, QC, Canada; Department of Oncology, Oncology Unit, ASST Lecco, Lecco, Italy; Breast Center, Women's Hospital, Marienhaus Hospital, Mainz, Germany; Klinikum Kulmbach—Frauenheilkunde und Geburtshilfe, Kulmbach, Germany; Department of Medical Oncology, Centre Jean Perrin, Clermont-Ferrand, France; Hochtaunus-Kliniken Bad Homburg, Women’s Hospital, Bad Homburg, Germany; Pôle Régional de Cancérologie, Centre Hospitalier Universitaire de Poitiers, Poitiers, France; Policlinico Gemelli, Rome, Italy; Health Sciences North, Sudbury, ON, Canada; Sozialstiftung Bamberg Klinikum Frauenklinik, Bamberg, Germany; Institut de Cancerologie de l’Ouest, Angers, France; Department of Gynecology and Obstetrics, Universitätsklinikum Münster, Münster, Germany; Royal Victoria Regional Health Centre, Barrie, ON, Canada; Department of Gynecology and Obstetrics, University Hospital Frankfurt, Germany; Rna Diagnostics, Inc, Toronto, ON, Canada; Rna Diagnostics, Inc, Toronto, ON, Canada; Policlinico Gemelli, Rome, Italy; Rna Diagnostics, Inc, Toronto, ON, Canada; Sunnybrook Health Sciences Centre, Toronto, ON, Canada

## Abstract

In previously reported retrospective studies, high tumor RNA disruption during neoadjuvant chemotherapy predicted for post-treatment pathologic complete response (pCR) and improved disease-free survival at definitive surgery for primary early breast cancer. The BREVITY (Breast Cancer Response Evaluation for Individualized Therapy) prospective clinical trial (NCT03524430) seeks to validate these prior findings. Here we report training set (Phase I) findings, including determination of RNA disruption index (RDI) cut points for outcome prediction in the subsequent validation set (Phase II; 454 patients). In 80 patients of the training set, maximum tumor RDI values for biopsies obtained during neoadjuvant chemotherapy were significantly higher in pCR responders than in patients without pCR post-treatment (*P* = .008). Moreover, maximum tumor RDI values ≤3.7 during treatment predicted for a lack of pCR at surgery (negative predictive value = 93.3%). These findings support the prospect that on-treatment tumor RNA disruption assessments may effectively predict post-surgery outcome, possibly permitting treatment optimization.

Patients with breast cancer undergoing neoadjuvant chemotherapy, with or without immune checkpoint inhibitors, often experience significant treatment side effects (1-6). Moreover, some patients with specific tumor subtypes derive little survival benefit from chemotherapy (1,2,7-10). Consequently, a real-time chemotherapy response assessment tool would be of significant value in managing patient care. This approach, termed “response-guided neoadjuvant chemotherapy” (11), may permit treatment escalation or de-escalation (12-15).

In the NCIC-CTG MA.22 clinical trial (16), high mid-treatment tumor ribosomal RNA (rRNA) fragmentation (RNA disruption) in patients with breast cancer was associated with both pathological complete response (pCR) and a greater than twofold increase in disease-free survival (DFS) after neoadjuvant chemotherapy (17). The level of tumor RNA disruption before chemotherapy was not predictive of treatment outcome. RNA disruption was quantified using the RNA disruption assay (RDA) (17), which computes an RNA disruption index (RDI) that is directly proportional to the degree of on-treatment RNA disruption (expressed as the ratio between abnormal rRNA fragments and normal 28S and 18S rRNA bands). Subsequent studies strongly supported the time- and dose-dependent association between exposure to various chemotherapy agents and both RNA disruption and tumor cell death in vitro (18,19) and in vivo (20). Tumor cell cultures with RDI values above 4.0 were consistently found to be nonviable, as measured by substantially decreased cell numbers, lack of cell replication after treatment (when returned to drug-free medium), and large increases in cell fragments with a subG1 DNA content (18). In a Her2+ breast cancer study, tumor RDI values after 1 cycle of neoadjuvant chemotherapy were twofold higher in those who achieved pCR compared with patients with residual disease (21).

The performance of the RDA to predict outcome from currently employed neoadjuvant chemotherapy regimens in patients with breast cancer is being further assessed in the BREVITY trial (Breast Cancer Response Evaluation for Individualized Therapy). BREVITY is a prospective 2-phase interventional study for women with invasive breast cancer of all subtypes and grades with T ≥1 cm who are receiving neoadjuvant chemotherapy and/or other standard of care drug regimens. Two biopsies are taken at each of 2 timepoints; 2 weeks after cycle 2 of chemotherapy (T1) and 2 weeks after cycle 1 of a second drug regimen (if administered) or at day 55 if multiple cycles of only 1 regimen were administered (T2). The training set (n = 80) had the primary objective of determining 2 RDI cut points that can quantify response to chemotherapy using RDA: Zone 1 (no response), Zone 2 (partial response), and Zone 3 (full response). Performance characteristics were optimized based on the negative predictive value (NPV) for Zone 1 and the positive predictive value (PPV) for Zone 3. In the training set, NPV and PPV were measured based on the absence or presence of pCR after surgery for individual patients in Zones 1 and 3, respectively. The validation set (n = 454, currently accruing patients) has the primary objective to validate the cut point of Zone 1 (established in the training set) by measuring the performance characteristic NPV for pCR. Possible differences in DFS for patients in Zones 1, 2, and 3 will also be assessed in the validation set as a secondary objective. Procedures followed complied with the ethical standards of the Helsinki Declaration and were approved by institutional review boards (including Advarra/ANSM/OCREB/BfArM and local ethics committees for all centers). All patients provided written informed consent before trial participation. The BREVITY trial protocol and statistical plan can be found in Supplementary Methods 1 (available online).

Freshly taken pseudonymized biopsies were stored and shipped in RNAlater™ fixative; total RNA was isolated from each tumor sample using RNeasy Mini Kits (Qiagen) and analyzed by capillary electrophoresis (Agilent 2100 Bioanalyzer). The RDI value was calculated for each sample from electropherogram data using a proprietary algorithm developed by Rna Diagnostics, Inc, and documented using an electronic case report form in a fully blinded fashion. In our analyses, the maximum RDI value (maximum level of chemo-responsiveness) was used for each patient to increase confidence that patients in Zone 1 were truly nonresponders. Other factors also impact clinical response, such as tumor heterogeneity, a switch in drug regimen, and/or increasing treatment time (where no switch in drug occurs). Additional details on trial design, eligibility criteria, sample size, statistical analyses, blinding, and primary and secondary endpoints can be found at https://clinicaltrials.gov/study/NCT03524430.

Analyzing the trial data for all training set patients (n = 80; Supplementary Table 1, available online), we report our assessment of the relationship between on-treatment tumor RNA disruption and pCR incidence after chemotherapy. Patients were accrued between September 2, 2020, and April 7, 2022, on the basis of the following tumor subtype distribution: HR+Her2+ (n = 16), HR+Her2- (n = 16), HR-Her2+ (n = 16) and HR-Her2- (n = 32). We then assessed the relationship between the maximum RDI value for each patient during treatment and pCR incidence (defined as ypT0-ypN0) at surgery.

Of 320 biopsies taken, 15 were not assessable (n/a) because of insufficient intact RNA remaining in the sample (4.7% of total). No patients were excluded from the training set because of multiple nonassessable biopsies. Seventy of 80 patients in the training set had a change in chemotherapy regimen between T1 and T2; 3 tumor subtypes (HR+Her2+, HR-Her2+, HR-Her2-) were represented within the patient group that did not have a change in drug regimen. Of the 10 patients included in the training set who had no change in therapy, 4 had an unplanned additional regimen given after the T2 biopsy.

The number of patients and mean maximum RDI values by subtype are shown in Table 1 for each of tumor stage, grade, histopathology, menopausal status, and pCR status after surgery. The pCR rate was 33.8% overall, with rates highest in Her2+ patients (37.5% and 43.7% for HR+Her2+ and HR-Her2+ tumors, respectively) and 37.5% for patients with HR-Her2- disease. Patients with HR+Her2- tumors had the lowest pCR rate (12.5%). Patients with pCR at surgery had significantly higher RDI values than patients without pCR (mean maximum RDI values of 11.3 ± 1.6 and 6.8 ± 0.6, respectively; *P* = .008; Mann-Whitney test). Median maximum tumor RDI values were also significantly different between pCR responders (9.0) and patients with residual disease (5.6) (*P* = .01 Mann-Whitney test).

**Table 1.** Baseline clinicopathologic characteristics and pCR status of patients in the training set^a^

	All		HR+Her2+		HR+Her2-		HR-Her2+		HR-Her2-	
	n = 80		n = 16		n = 16		n = 16		n = 32	
	n (%)	RDI	n (%)	RDI	n (%)	RDI	n (%)	RDI	n (%)	RDI
**Tumor stage**										
I	10 (12.5)	8.7 ± 1.8	2 (12.5)	14.5 ± 8.1	1 (6.2)	5.1	1 (6.2)	8.2	6 (18.8)	7.5 ± 1.7
IIA	34 (42.5)	7.8 ± 0.9	5 (31.2)	11.0 ± 1.8	6 (37.5)	6.2 ± 2.0	7 (43.8)	9.2 ± 1.6	16 (50.0)	6.7 ± 1.5
IIB	16 (20.0)	7.4 ± 1.4	2 (12.5)	19.4 ± 2.6	4 (25.0)	2.9 ± 1.0	4 (25.5)	7.6 ± 1.6	6 (18.8)	6.2 ± 1.1
IIIA	13 (16.2)	8.0 ± 1.5	5 (31.2)	11.1 ± 2.9	3 (18.9)	7.6 ± 2.7	2 (12.5)	8.4 ± 0.6	3 (9.4)	2.9 ± 1.2
IIIB	3 (3.8)	7.7 ± 1.4	1 (6.2)	5.1	1 (6.2)	8.3	1 (6.2)	9.7	0	n/a
No information	4 (5.0)	17.3 ± 7.3	1 (6.2)	7.5	1 (6.2)	10.0	1 (6.2)	12.7	1 (3.1)	38.9
**Grade**										
2	35 (43.8)	9.8 ± 1.2	11 (68.8)	11.2 ± 2.0	9 (56.2)	6.2 ± 1.1	7 (43.8)	8.9 ± 1.1	8 (25.0)	12.7 ± 4.2
3	42 (52.5)	6.8 ± 0.7	4 (25.0)	11.3 ± 2.0	7 (43.8)	5.6 ± 1.9	9 (56.2)	8.9 ± 1.3	22 (68.8)	5.5 ± 0.9
No information	3 (3.8)	12.4 ± 4.9	1 (6.2)	22.0	0	n/a	0	n/a	2 (6.2)	7.6 ± 1.4
**Histopathology**										
Infiltrating ductal	67 (83.8)	8.4 ± 0.8	13 (81.2)	12.4 ± 1.7	14 (87.5)	6.3 ± 1.1	14 (87.5)	8.2 ± 0.6	26 (81.2)	7.8 ± 1.6
Infiltrating lobular	4 (5.0)	10.0 ± 2.2	2 (12.5)	12.1 ± 3.2	1 (6.2)	5.2	0	n/a	1 (3.1)	10.1 ± 1.6
Inflammatory	1 (1.2)	9.7	0	n/a	0	n/a	1 (6.2)	9.7	0	n/a
Poorly differentiated	1 (1.2)	3.8	0	n/a	0	n/a	0	n/a	1 (3.1)	3.8
Not specified	7 (8.8)	6.4 ± 2.0	1 (6.2)	5.4	1 (6.2)	2.1	1 (6.2)	17.8	4 (12.5)	5.0
**Menopausal status**										
Premenopausal	33 (41.2)	8.0 ± 1.0	6 (37.5)	14.8 ± 2.7	8 (50.0)	4.6 ± 0.8	6 (37.5)	10.4 ± 1.9	13 (40.6)	5.8 ± 1.4
Perimenopausal	2 (2.5)	5.5 ± 0.9	1 (6.2)	4.6	0	n/a	0	n/a	1 (3.1)	6.4
Postmenopausal	43 (53.8)	8.7 ± 1.0	8 (50.0)	10.1 ± 1.9	7 (43.8)	8.1 ± 1.8	10 (62.5)	8.0 ± 0.6	18 (56.2)	8.6 ± 2.1
Unknown	2 (2.5)	8.8 ± 7.8	1 (6.2)	16.7	1 (6.2)	1.0	0	n/a	0	n/a
**pCR status**										
pCR	27 (33.8)	11.3 ± 1.6	6 (37.5)	14.5 ± 3.1	2 (12.5)	4.8 ± 0.8	7 (43.8)	9.9 ± 1.6	12 (37.5)	11.7 ± 2.9
No pCR	53 (66.2)	6.8 ± 0.6	10 (62.5)	10.4 ± 1.6	14 (87.5)	6.1 ± 1.2	9 (56.2)	8.1 ± 0.9	20 (62.5)	4.9 ± 0.8
All	80 (100)	8.3 ± 0.7	16 (100)	11.9 ± 1.5	16 (100)	5.9 ± 1.0	16 (100)	8.9 ± 0.8	32 (100)	7.4 ± 1.3

a Number of patients and mean maximum RNA disruption index (RDI) values are shown for all patients in the training set and by subtype separated by tumor stage, grade, histopathology, and menopausal or pathologic complete response (pCR) status. Maximum RDI value is the highest RDI value for each patient obtained from 4 biopsies taken at 2 timepoints.

Maximum RDI cut-point values were selected to separate patients in Zones 1, 2, and 3, based on optimizing the NPV for pCR in Zone 1 and the PPV for pCR in Zone 3. The cut point between Zones 1 and 2 was selected at RDI 3.7, where 15 patients (26% of patients without pCR) were captured in Zone 1 (RDI ≤3.7) at an NPV for pCR of 93.3% (Figure 1, A). The cut point between Zones 2 and 3 was selected at RDI 10.0; Zone 3 (RDI >10.0) captured 44% of patients with a pCR. Although the PPV for pCR in Zone 3 was only 52% (Figure 1, B), prior retrospective studies (MA.22 (17) and NeoAva (22) clinical trials) suggested that high tumor RNA disruption is a better predictor of DFS than pCR. Patients with high on-treatment tumor RNA disruption in the MA.22 trial (n = 38) were almost 5-fold higher in number than pCR responders (n = 8). With or without a pCR, patients in Zone 3 had very similar DFS durations to pCR responders (17). Consequently, high tumor RDI values during treatment may be superior to pCR at surgery in predicting survival after neoadjuvant chemotherapy. Recent meta-analyses have questioned the utility of pCR to predict outcome after neoadjuvant chemotherapy across all tumor subtypes (23-25). The association between high tumor RNA disruption on-treatment and improved DFS will be assessed in the BREVITY validation set with the collection of 3- and 5-year survival data. The utility of RDA for predicting treatment outcome in specific breast tumor subtypes will also be assessed as a secondary objective in the larger validation set.

**Figure 1.**
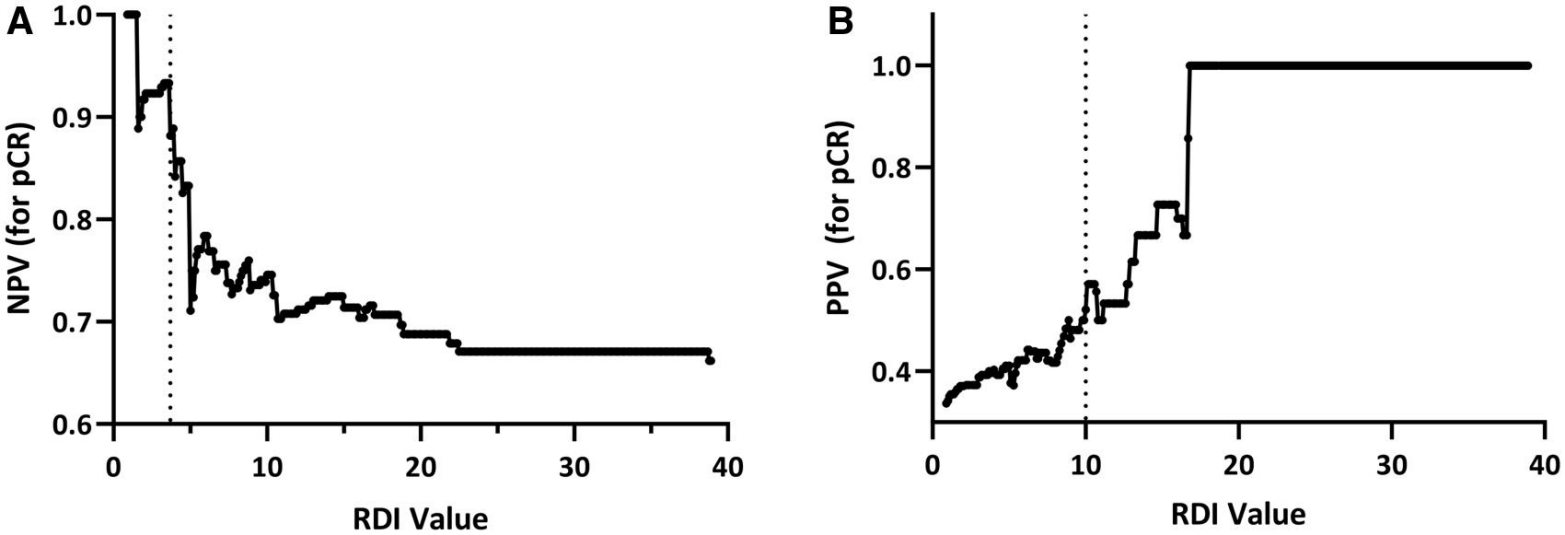
**A)** Plot of negative predictive value (NPV) for pathologic complete response (pCR) calculated for each maximum RNA disruption index (RDI) value; Zone 1 defines patients with maximum RDI values ≤3.7. **B)** Plot of positive predictive value (PPV) for pCR for each maximum RDI value; Zone 3 defines patients with maximum RDI values >10.0.

In summary, the primary objective of the training set, establishment of a Zone 1 cutoff associated with an NPV for pCR, was met. The training set data show that low on-treatment tumor RNA disruption (RDI ≤3.7) is strongly associated with a lack of pCR at surgery. Validating the performance characteristics of the RDI Zone 1 cut point is the primary objective of the BREVITY validation set (accrual ongoing); secondary objectives include assessments of pCR prevalence and DFS across tumor subtypes. Tumor RNA disruption measurements can be rapidly and easily performed in early-stage disease. In contrast, current circulating tumor DNA (ctDNA) approaches require pretreatment genomic DNA sequencing of normal and tumor breast tissue from each patient to identify and quantify tumor-specific ctDNAs in blood at various times before, during, or after neoadjuvant chemotherapy (26,27). Often, ctDNA is undetectable in EBC (28), thus limiting the approach or restricting its use to patients with high-risk or later-stage disease (26). Therefore, the ability of the RDA to rapidly identify nonresponding patients during chemotherapy could be of great value to physicians in making further treatment decisions.

## Supplementary Material

pkad107_Supplementary_Data

## Data Availability

The full dataset underlying this article can be found in Supplementary Table 1 (available online).
