# Supplementary material for: Low RNA disruption during neoadjuvant chemotherapy predicts pathologic complete response absence in patients with breast cancer
Source: JNCI Cancer Spectr. 2023 Dec 19;8(1):pkad107. doi: 10.1093/jncics/pkad107 (PMC10765091; doi:10.1093/jncics/pkad107)
Supplement: pkad107_Supplementary_Data [file pkad107_supplementary_data.pdf]

SUPPLEMENTARY MATERIALS

Supplementary Methods 1: BREVITY Protocol & Stats Plan

BREVITY

Breast Cancer **R**esponse **EV**aluation for **I**ndividualized **T**herap**Y**

**Study Title:**	RNA Disruption Assay (RDA) - **Bre**ast cancer **R**esponse **EV**aluation for **I**ndividualized **T**herap**Y** (*BREVITY*)
**Study Number:**	RnaDx -BRV-BC-01
**Version Number /Date:**	Protocol Version 18 (USA) - March 2022
**Study Sponsor:**	Rna Diagnostics Inc. (« Rna Diagnostics »)<br>*21 St. Clair Avenue east, Suite 701, Toronto, Ontario, Canada M4T 1L9*
**Co- Chief Investigators / International Principal Investigators:**	*Name: Dr. Maureen Trudeau*<br>*Institution: Sunnybrook Health Sciences Centre, Toronto, Ontario, Canada.*<br>*Email: Maureen.Trudeau@sunnybrook.ca*<br>*Phone: +1 416-480-4270*<br><br>*Name: Dr. Daniele Generali*<br>*Institution: Department of Medical Surgery and Health Sciences, University of Trieste and Ospedale Cremona, Italy.*<br>*Email: daniele.generali@gmail.com*
**Principal Investigators (by Country)**	*Name: Dr. Foluso Ademuyiwa*<br>*Institution: Division of Medical Oncology, Washington University School of Medicine in St Louis, MO, USA.*<br><br>*Name: Dr. Daniele Generali*<br>*Institution: Department of Medical Surgery and Health Sciences, University of Trieste and Ospedale Cremona, Italy.*<br><br>*Name: Dr. Maureen Trudeau*<br>*Institution: Sunnybrook Health Sciences Centre, Toronto, Ontario, Canada.*<br><br>*Name: Dr.Thierry Petit*<br>*Institution: Départment d'Oncologie médicale, Centre Paul Strauss, Strasbourg, France.*<br><br>*Name: Dr. Eva Ciruelos*<br>*Institution: Hospital U. 12 de Octubre, Madrid, Spain*<br><br>*Name: Dr. Joke Tio (for BREVITY-02 in Germany)*<br>*Institution: Universitätsklinikum Münster, Germany.*

**Statistician**	*Name: Carolin Junglen*<br>*AMS Advanced Medical Services GmbH*<br>*Am Exerzierplatz 2*<br>*68167 Mannheim, Germany*

PROTOCOL AUTHORISATION

**Study Title:**	RNA Disruption Assay (RDA) - **Br**east cancer **R**esponse **EV**aluation for **I**ndividualized **T**herap**Y** (*BREVITY*)
**Version/ date:**	Version 18 (USA) / March, 2022
**Study Number:**	RnaDx -BRV-BC-01

A review of the protocol has been completed and is understood and approved by the following:

Principal Investigators by Country	Signature	Date (dd-MON-yyyy)

Sanaa Noubir<br>VP, Clinical Development	SN	28 / Mar / 2022
Sponsor<br>Rna Diagnostics Inc.	Signature	Date (dd-Mon-yyyy)

TABLE OF CONTENTS

LIST OF ABBREVIATIONS **6**

1. STUDY SYNOPSIS **7**

2. BACKGROUND AND RATIONALE **11**

2.1 Background 11

2.2 Rationale 12

2.3 Hypothesis 12

2.4 RDA Test Components and Description 13

3. STUDY OBJECTIVES and ENDPOINTS **13**

3.1 Primary and secondary objectives 13

3.2 Endpoints definitions 14

4. PATIENT POPULATION **14**

4.1 Patient selection criteria 14

4.1.1 Inclusion criteria 15

4.1.2 Exclusion criteria 15

4.2 Number of patients 15

4.3 Anticipated enrolment period 16

4.4 Duration of patient participation 17

4.5 BREVITY study (in combination with BREVITY-02 in Germany) anticipated timelines for phase 1 and phase 2 17

4.6 Withdrawal criteria and procedures 17

4.7 Estimation of study completion 17

5. STUDY DESIGN **17**

5.1 Type of study design 17

5.2 Schematic of BREVITY study design 18

5.3 Timing of sample collection 19

5.4 Study parameters and enrolment procedure 19

5.4.1 Screening 20

5.4.2 Consenting and registration 20

5.4.3 Sample and data collection 20

5.4.4 Follow-up 21

6. SAFETY ASSESSMENT **21**

6.1 Safety assessments considerations 21

6.1.1 Physical examination 21

6.1.2 Clinical safety laboratory assessment 21

6.2 Reporting of serious adverse events 22

6.3 Risk – benefit assessment and COVID-19 related risk mitigation measures 22

7. INFORMATION/ PROCEDURES ON SAMPLES **23**

7.1 Sample processing 23

7.2 Storage and transport of samples 23

7.3 Facilities to be used and contact details 25

8. IDENTIFICATION OF SOURCE DATA/ DATA TO BE ENTERED ON CRFS **25**

9. STATISTICAL CONSIDERATIONS 25
9.1 RDI scores 25
9.2 Rationale of statistical design 26
9.3 Primary analysis 27
9.4 Determination of sample size 28
9.5 Analysis populations 29
9.6 Statistical methods 29
9.7 Handling of missing values 31
10. ADMINISTRATIVE RESPONSIBILITIES 31
10.1 Good clinical practice 31
10.2 Ethical considerations 31
10.3 Patient information and informed consent 32
10.4 Patient confidentiality 32
10.5 Steering Committee (SC) 32
10.6 Protocol violation (or deviations) and non-compliance 32
10.7 Monitoring, auditing and inspecting 33
10.8 Premature closure of the study 33
10.9 Data quality assurance 34
10.10 Record retention 34
10.11 Publication policy 34
10.12 Indemnity 35
10.13 Patient expense reimbursement 35
10.14 Data privacy and protection 35
11. APPENDICES 36
APPENDIX A: REFERENCES 36
APPENDIX B: SAMPLE COLLECTION TIME POINTS, PROCESSING, STORAGE AND TRANSPORT 38
APPENDIX C: INVESTIGATOR AGREEMENT (mandatory) 43

LIST OF ABBREVIATIONS

Abbreviation	Full Form
BREVITY	***Br**east Cancer **R**esponse **EV**aluation for **I**ndividualized **T**herap**Y***
BREVITY-02	***Breast Cancer Response EValuation for Individualized TherapY – 02 (in Germany)***
RDA	*RNA Disruption Assay*
RDI	*RNA Disruption Index*
rRNA	*Ribosomal Ribonucleic Acid*
LABC	*Locally Advanced Breast Cancer*
pCR	*Pathological Complete Response*
RCB	*Residual Cancer Burden*
DFS	*Disease Free Survival*
OS	*Overall Survival*
HR, ER, PR, HER2, TNBC	*Estrogen and/or Progesterone Receptor, Human Epidermal Growth Factor Receptor 2, Triple Negative Breast Cancer*
NPV	*Negative Predictive Value*
PPV	*Positive Predictive Value*
SoC	*Standard of Care*
SC	*Steering Committee*
18F-FDG PET/CT	*18F-fluorodeoxyglucose Positron Emission Tomography / Computerized Tomography*
MA.22 or MA.22 NCIC-CTG Trial	*MA.22 National Cancer Institute of Canada - Clinical Trials Group (NCIC-CTG) Trial - A neoadjuvant trial of locally advanced breast cancer patients*
FNA	*Fine Needle Aspiration*
FFPE blocks	*Formalin-Fixed Paraffin-Embedded blocks*
CRF	*Case Report Form*
ICH-GCP	*International Conference on Harmonisation – Good Clinical Practice*
IRB/IEC	*Institutional Review Board / Independent Ethics Committee*
SAE	*Serious Adverse Event*
EDC	*Electronic Data Capture*
MCAR	*Missing Completely at Random*
MAR	*Missing at Random*
QC	*Quality Control*
SDV	*Source Document Verification*
SDR	*Source Data Review*

1. STUDY SYNOPSIS

**Study Title/ Short Name**	RNA Disruption Assay (RDA) - **Br**east cancer **R**esponse **EV**aluation for **I**ndividualized **T**herap**Y** (*BREVITY*)
**Study Objectives and Endpoints:**	

**Primary Objectives**	**Description**	**Endpoints**
i.	1. Phase 1: To determine 2 RDI cut-offs to have a diagnostic test optimized in terms of predictive values.<br>2. Phase 2: To establish performance characteristics for the first cut-off (test result “zone 1”) in terms of the NPV (*Zone 1 represents the test’s main clinical utility and could be useful as an aid in optimizing neoadjuvant treatment*).	NPV, PPV

**Secondary Objectives**	**Description**	**Endpoints**
i.	To assess the diagnostic test’s NPV in cancer subgroups by subtype *e.g.:*<br>• HR+/HER2-<br>• HR-/HER2+<br>• HR+/HER2+<br>• HR-/HER2-	NPV
ii.	To assess the diagnostic test’s PPV in HER2+ patients.	PPV
iii.	To assess and compare pCR prevalence and Residual Cancer Burden RCB class in zones 1-3 for cancer subgroups by subtype *e.g.:*<br>• all patients<br>• HR-/HER2-<br>• HR-/HER2+<br>• HR+/HER2+<br>• HR+/HER2-<br>• all HER2+<br>• all HER2+ and HR-/HER2- combined	pCR, RCB class at surgery
iv.	To assess and compare DFS in zones 1-3 for cancer subgroups by subtype *e.g.*:<br>• all patients<br>• HR-/HER2-<br>• HR+/HER2+<br>• HR+/HER2-<br>• HR-/HER2+<br>• all HER2+<br>• HR-/HER2- and all HER2+ combined<br>• HR-/HER2+ and Luminal B	DFS
v.	To test for association between cancer subgroups by subtype and test result.	Test result (zones 1-3)

<table>
<tr>
<td></td>
<td>
<table>
<tr><td>vi.</td><td>To compare RDA results to other potential biomarker results such as the marker of proliferation Ki67 and tumour grade (if results are available).</td><td></td></tr>
</table>
</td>
</tr>
<tr>
<td>Patient Population</td>
<td>
The population consists of patients diagnosed with invasive breast cancer and scheduled to receive neoadjuvant chemotherapy as part of standard of care treatment. RDA is presently in an experimental stage and clinicians will not use the RDA results to manage patients’ therapy in this study. Throughout the program, patients will receive standard of care neoadjuvant chemotherapy treatments including taxanes, anthracyclines or other targeted drugs and drug combinations as prescribed based on the investigators’ / clinicians’ choice. Adjuvant therapies (e.g. radiotherapy, hormonal treatment … etc.) may be prescribed to patients according to standard of care and independently of the RDI score results.
Inclusion Criteria

Women aged at least 18 years;
Patients must be able to provide informed consent and sign the informed consent form to participate in the RDA study before any study procedure starts;
Newly diagnosed clinical stage I, II or III breast cancer with complete surgical excision of the breast cancer after neoadjuvant therapy as the treatment goal;
Tumour size at least 1 cm in one dimension by clinical or radiographic exam (WHO criteria);
Must have histological confirmation of invasive breast cancer of any subtype or grade;
Patient is scheduled for neoadjuvant chemotherapy +/- antibodies and +/- other drugs according to Standard of Care;
Patient is willing to have 2 research core needle biopsies (for RDA) taken at 2 collection timepoints during neoadjuvant chemotherapy treatment.

Exclusion Criteria

Patient who has had prior local (i.e. surgery or radiotherapy) or systemic (i.e. endocrine or cytotoxic) therapy for the current breast cancer;
Participation in another interventional clinical trial with concurrent treatment with experimental drugs to treat the current breast cancer during the period of neoadjuvant therapy (from diagnosis until surgery);
Stage IV breast cancer;
Bilateral or multicentric breast cancer;
Prior malignant disease except curatively treated in-situ malignancies;
Concurrent pregnancy;
Breast feeding woman;
Concurrent medical, psychiatric or addictive disorders that may limit the ability to give informed consent or complete the trial;
Reasons indicating risk of poor compliance with study procedures;
Patient not able to consent.

</td>
</tr>
</table>

	**Number of Patients:**

The study aims to enrol approximately 594 patients (in combination with the BREVITY-02 study in Germany) in order to achieve an accrual of 534 fully evaluable patients which is the number required to adequately power the trial. This accounts for an estimated 10% drop out rate. 80 fully evaluable patients are required for the training set (phase 1) while 454 fully evaluable patients will be required in phase 2 for the validation set. Patient distribution based on breast cancer subtype will be controlled.

The patient will be approached for potential accrual and consent after a diagnosis is made and the patient's cancer subgroup by subtype is determined. This patient recruitment approach will allow to know a patient's cancer subgroup by subtype in advance and to control the recruitment to each subgroup. Recruitment will be open simultaneously for all cancer subgroups by subtype. When the number required internationally for a patient subgroup (e.g. 119 HR+/Her2- patients) is reached, the data management and monitoring team will instruct all participating sites to stop the recruitment of patients belonging to this specific subgroup. This monitoring process will prevent any excess accruals and ensure that only the required accrual numbers for each cancer subgroup by subtype are achieved. In case a patient signed the informed consent form on the same day when the required patient number was reached, the patient can still participate in the study.

The competitive recruitment will be closed when the respective numbers of patients to enrol from each cancer subgroup by subtype are achieved as shown in the following tables:

Cancer Subgroups by Subtype	Percentage of patients	Fully evaluable patients	Patients to be enrolled (based on DO rate)
HR+/HER2-	20%	107	119
HR-/HER2-	40%	213	237
HR+/HER2+	20%	107	119
HR-/HER2+	20%	107	119

Required patients for Phase I (target of 80 fully evaluable patients):

Cancer Subgroup by Subtype	Percentage of patients	Fully evaluable patients	Patients to be enrolled (based on DO rate)
HR+/HER2-	20%	16	18
HR-/HER2-	40%	32	36
HR+/HER2+	20%	16	18
HR-/HER2+	20%	16	18

Analysis will be performed when the minimum number of patients is reached for every cancer subgroup by subtype. Recruitment per subgroup of cancer subtypes will not be closed until target number of patients per subgroup is reached for phase 2.

Required patients for Phase II (target of 454 fully evaluable patients):

Cancer Subgroup by Subtype	Percentage of patients	Fully evaluable patients	Patients to be enrolled (based on DO rate)
HR+/HER2-	20%	91	101
HR-/HER2-	40%	181	201
HR+/HER2+	20%	91	101
HR-/HER2+	20%	91	101

Recruitment will be stopped when the required numbers of patients for each cancer subgroup by subtype are reached.

The study aims to enrol approximately 594 patients (in combination with the BREVITY-02 study in Germany).

The target patient enrolment by country is as follows: 45 patients in 5-6 centres in the US, 82 patients in 5-6 centres in Canada, 145 patients in 12-15 centres in Italy, 100 patients in up to 15 sites in France, 100 patients in up to 15 centres in Spain, in addition to 150 patients in up to 15 German centres (in accordance with the protocol of the BREVITY-02 study RnaDx-BRV-BC-02). These target patient accrual numbers may vary (can be increased or decreased) depending on the patient accrual speed in each participating country and patient accrual will be stopped when the required total number of fully evaluable patients will be achieved globally.

The protocol of the BREVITY-02 study in Germany is in full concordance with the protocol of the international BREVITY study. All data from BREVITY-02 will be amalgamated with international BREVITY and analysed as a unified data set.

Study Design:

Study type: This is a prospective diagnostic performance two-phase multicentre study.

Samples: 2 core needle biopsy specimens will be taken at each biopsy collection time point for RDA analysis during chemotherapy.

Timing of biopsy collection:

Table 1. Biopsy Collection Time Points		
Biopsy	*1st core needle biopsy for RDA (2 specimens)*	*2nd core needle biopsy for RDA (2 specimens)*
Biopsy timing by type of drug schedule	*35 days +/- 4 days*	***If no change of drugs:***<br>*55 days +/- 5 days*<br><br>***If there is a change of drugs:***<br>*2 to 3 weeks after start of new drugs as follows depending on drug regimen:*<br>• *3-weekly: at 16 days +/- 2 days*<br>• *Bi-weekly: at day of 2nd dose preferably before drug admin.*<br>• *Weekly: at day of 4th dose preferably before drug admin.*

	**Sample collection, storage and shipment details:** Each of the 2 biopsy specimens collected for RDA testing will be immediately placed in a separate collection tube containing 1ml RNA fixative (RNAlater® Tissue Collection Solution supplied by Rna Diagnostics). The collection tubes will be identified with the clinical site ID number, subject ID number, date of biopsy and fixative lot number. The tubes should be inverted 3 times then kept between 15-25°C temperature overnight before being stored in a -20°C or a -80°C freezer. All tubes containing biopsies will be stored such that the temperature is -10°C or less until the day of collective shipment which will be either when about 10 patients have been accrued in the same centre or at day 90 (biopsies should not be kept in the freezer for longer than 90 days). The CRO/SMOs will notify the centres on a quarterly basis. On the shipping day (preferably a Monday), patient Cryo transport boxes will be placed in a clear plastic bag that goes in an insulated (Styrofoam) box with 4 pre-frozen cold packs along with a temperature logger. The box will be sent to the Rna Diagnostics laboratory in Canada using the courier services information provided by Rna Diagnostics. Please refer to **Appendix B** for detailed core biopsy collection, storage and shipping guidelines.<br>Rna Diagnostics will perform the RDA within 2 weeks from the reception of the biopsy specimens.<br>The remaining RNA isolated from the tumour biopsies will be stored in the RNA diagnostics laboratory in a -20°C or -80°C for further research and for up to 10 years before being destroyed.<br>FFPE blocks of biopsy taken at pre-therapy for diagnosis saved in the participating centre facility may also be requested by Rna Diagnostics to be used in further analysis, provided that sufficient material will remain in the participating centre for further diagnostics.<br><br>**Follow-up duration:** Patients will be followed up for 60 months from the date of enrolment in the study.

2. BACKGROUND AND RATIONALE

2.1 Background

RDA is a molecular test based on the analysis of RNA disruption, a qualitative and quantitative alteration of the ribosomal ribonucleic acid (rRNA) of tumour cells that correlates with chemotherapy response in primary breast cancer. In the MA.22 NCIC Clinical Trials Group (NCIC-CTG) neoadjuvant trial of locally advanced breast cancer patients, the trial from which RDA was developed, the extent of RNA disruption during treatment, as assessed by a proprietary algorithm and expressed by an RNA Disruption Index (RDI), stratifies patients' response into 3 response zones: non-response (zone 1), partial-response (zone 2) and response (zone 3). Survival results from the patients involved in the MA.22 trial showed significantly superior 5-year Disease Free Survival (DFS) Kaplan Meier curves for patients with RDA in zone 3 compared to patients with RDA in zones 1 or 2 [1].

In preclinical studies, a dose and time dependent increase in rRNA disruption is observed as a result of drug treatments in sensitive tumour cell lines (absent in drug resistant tumour cell lines)[2].

In vitro studies have also demonstrated that rRNA disruption occurs in response to a variety of chemotherapeutic drugs, as well as irradiation.

The current study aims to achieve clinical validation of RDA based on biopsies performed during treatment as early as 31-39 days after chemotherapy initiation which would greatly enhance RDA's potential clinical utility. Recent additional clinical studies have indicated that lack of pCR and clinical progression is preferentially associated with absent or minimal RNA disruption measured as early as 20 days following the first cycle of neoadjuvant chemotherapy [3]. Furthermore, in a window of opportunity study using various drugs, RNA disruption has been observed as early as 14 days post neoadjuvant treatment initiation [4]. Studies using 18F-FDG PET/CT have also shown that metabolic changes to breast tumours are measurable and can be correlated to pCR as early as 14 days after the start of treatment [5-7] providing further evidence that molecular assessments of tumour response as early as 31-39 days are not too early.

The clinical utility of RDA is greater the earlier during treatment it can be measured. Predicting response to drugs as early as 14 days after treatment initiation could allow treatment adaption after only one cycle of chemotherapy. However, current reassessment of the RDI score range at T1 (14 days) has shown a distribution of RDI scores too narrow to provide an adequate separation between the response zones. Recent analysis of RDI scores from an unpublished study have shown that up to 45% of patients show a significant increase of RDI scores between cycle 1 and cycle 2 indicating a relatively slower response to the drugs in these patients. We hypothesize that RDI measured after 2 cycles of drug treatment (35 +/- 4 days) will allow an accurate RDA assessment for all patients, including patients who are slow responders. At the same time RDA measured as early as 31-39 days can provide an opportunity to adapt treatment and avoid cycles of an ineffective therapy. Escalation / de-escalation treatment strategies can be implemented effectively after 31 days or 2 cycles of chemotherapy.

2.2 Rationale

Neoadjuvant therapy is an increasingly common mode of chemotherapy in clinical practice and has been included as a standard treatment approach to render operable non-resectable breast cancers and evaluate *in-vivo* response to drugs. There is some evidence that identifying non-responders early in neoadjuvant treatment and offering alternative agents (response-guided or adaptive therapy) increased pCR rates and/or survival while minimizing morbidity [8]. Response guided neoadjuvant therapy was also shown to result in an Incremental Cost Effectiveness Ratio (ICER) that was cost effective [9].

Differentiating non-responders to chemotherapy from responders with reliable guidance from well-validated tests is crucial to the success of response-guided therapy. Non response to chemotherapeutics is common in primary breast cancer patients; therefore, implementation of adaptive therapy to avoid ineffective treatment would be advantageous. Increased accuracy in response assessment can translate into better outcomes and added cost effectiveness. Also, early reliable assessment is most useful for a timely adaptation of the treatment. The current study will support clinical use of RDA as a response assessment tool that uses tumour core biopsies taken as early as 31-39 days after the initiation of neoadjuvant treatment.

2.3 Hypothesis

The study will validate – in primary breast cancer patients undergoing neoadjuvant chemotherapy – RDA's diagnostic test accuracy and investigate its performance as a predictive marker of pathological complete response and disease-free survival. In this study, hypotheses to be verified relate to RDA's ability to predict chemotherapy effectiveness in terms of pCR / non pCR and enhanced disease-free survival based on tumour biopsies taken as early as 31-39 days after chemotherapy initiation.

2.4 RDA Test Components and Description

The RNA Disruption Assay is an in-vitro diagnostic test being developed to be provided as a diagnostic testing service. RDA is based on the analysis of RNA disruption, a qualitative and quantitative alteration of the ribosomal ribonucleic acid (rRNA) of tumour cells that correlates with drug response in primary breast cancer. RDA is performed in a tumor core biopsy (2 specimens) obtained during chemotherapy +/- targeted drugs +/- other drugs. RNA is isolated using commercially available kits using a column based manual RNA isolation system, the RNA is analyzed using an Agilent 2100 Bioanalyzer and RDI values are determined from the electropherogram data using a proprietary algorithm coded in MatLab (Mathworks). Sample tracking from receipt at Rna Diagnostics' laboratory through RNA storage is performed using FreezerWorks software (Dataworks).

All components used in the RDA testing process are purchased from suppliers and qualified through the quality control processes. BREVITY samples will be processed under GCLP which was granted on September 27, 2018.

Although predicted to be minimal, any risk related to the use of core needle biopsy during treatment in the BREVITY trial, will be mitigated by implementing biopsy safety assessments and serious adverse events reporting and follow-up as outlined in section 6.

3. STUDY OBJECTIVES and ENDPOINTS

3.1 Primary and secondary objectives

Primary Objectives	Description	Endpoints
i.	1. Phase 1: To determine 2 RDI cut-offs to have a diagnostic test optimized in terms of predictive values.<br>2. Phase 2: To establish performance characteristics for the first cut-off (test result "zone 1") in terms of the NPV (*Zone 1 represents the test's main clinical utility and could be useful as an aid in optimizing neoadjuvant treatment*).	NPV, PPV

Secondary Objectives	Description	Endpoints
i.	To assess the diagnostic test's NPV in cancer type subgroups by subtype:<br>• HR+/HER2-<br>• HR-/HER2+<br>• HR+/HER2+<br>• HR-/HER2-	NPV
ii.	To assess the diagnostic test's PPV in HER2+ patients.	PPV
iii.	To assess and compare pCR prevalence and Residual Cancer Burden RCB class in zones 1-3 for the following cancer subgroups by subtype:<br>• all patients<br>• HR-/HER2-<br>• HR-/HER2+<br>• HR+/HER2+<br>• HR+/HER2-<br>• all HER2+<br>• all HER2+ and HR-/HER2- combined	pCR, RCB class at surgery

iv.	To assess and compare DFS in zones 1-3 for the following cancer subgroups by subtype:<br>• all patients<br>• HR-/HER2-<br>• HR+/HER2+<br>• HR+/HER2-<br>• HR-/HER2+<br>• all HER2+<br>• HR-/HER2- and all HER2+ combined<br>• HR-/HER2+ and Luminal B	DFS
v.	To test for association between cancer subgroups by subtype and test result.	Test result (zones 1-3)
vi.	To compare RDA results to other potential biomarker results such as the marker of proliferation Ki67 and tumour grade (if results are available).	

3.2 Endpoints definitions

Endpoint	Definition
NPV	Negative predictive value, defined as<br>**P**[no pCR | zone i]<br>(probability of <u>not</u> achieving pCR given test result "zone i", for i = 1, 2, 3)
PPV	Positive predictive value, defined as<br>**P**[pCR | zone i]<br>(probability of achieving pCR given test result "zone i", for i = 1, 2, 3)
pCR	Both pCR definitions (ypT0 ypN0: absence of invasive cancer and in situ cancer in the breast and axillary nodes AND ypT0/is ypN0: absence of invasive cancer in the breast and axillary nodes irrespective of carcinoma in situ) which have been shown to be associated with long-term outcome[10] will be considered in the endpoint correlative analysis.
DFS	Disease-free survival defined as timespan between diagnosis and first event of progression or death. Events considered as progression are:<br>• presence by imaging of any new local relapse after surgery<br>• presence of any metastasis observed by imaging after surgery
Test result	Test result is one of three categories<br>• zone 1<br>• zone 2<br>• zone 3<br>which result from the division of RDI parameter space by the two cut-offs determined in the phase 1 primary objective.

4. PATIENT POPULATION

The population consists of patients diagnosed with invasive breast cancer and scheduled to receive neoadjuvant chemotherapy as part of standard of care treatment. RDA is presently in an experimental stage and clinicians will not use the RDA results to manage patients' therapy in this study.

4.1 Patient selection criteria

4.1.1 Inclusion criteria

Female patients must fulfill all of the following criteria to be included in the study:

- Women aged at least 18 years;
- Patients must be able to provide informed consent and sign the informed consent form to participate in the RDA study before any study procedure starts;
- Newly diagnosed clinical stage I, II or III breast cancer with complete surgical excision of the breast cancer after neoadjuvant therapy as the treatment goal;
- Tumour size at least 1 cm in one dimension by clinical or radiographic exam (WHO criteria);
- Must have histological confirmation of invasive breast cancer of any subtype or grade;
- Patient is scheduled for neoadjuvant chemotherapy +/- antibodies (targeted therapy such as trastuzumab) and +/- other drugs according to Standard of Care;
- Patient willing to have 2 research core needle biopsies (for RDA) taken at 2 collection timepoints during neoadjuvant chemotherapy treatment.

4.1.2 Exclusion criteria

Patients with one or more of the following criteria will be excluded from the study:

- Patients who have had prior local (i.e. surgery or radiotherapy) or systemic (i.e. endocrine or cytotoxic) therapy for the current breast cancer;
- Participation in another interventional clinical trial with concurrent treatment with experimental drugs to treat the current breast cancer during the period of neoadjuvant therapy (from diagnosis until surgery);
- Stage IV breast cancer;
- Bilateral or multicentric breast cancer;
- Concurrent medical, psychiatric or addictive disorders that may limit the ability to give informed consent or complete the trial;
- Prior malignant disease except curatively treated in-situ malignancies;
- Concurrent pregnancy;
- Breast feeding woman;
- Reasons indicating risk of poor compliance with study procedures;
- Patients not able to consent.

4.2 Number of patients

The study aims to enrol approximately 594 patients (in combination with BREVITY-02 in Germany) in order to achieve an accrual of 534 fully evaluable patients which is the number required to adequately power the trial. This accounts for an estimated 10% drop out rate. 80 fully evaluable patients are required for the training set (phase 1) while 454 fully evaluable patients will be required in phase 2 for the validation set.

Patient distribution based on breast cancer subtype will be controlled.

The patient will be approached for potential accrual and consent after a diagnosis is made and the patient's cancer subgroup by subtype is determined. This patient recruitment approach will allow to know a patient's cancer subgroup by subtype in advance and to control the recruitment to each subgroup. Recruitment will be open simultaneously for all cancer subgroups by subtype. When the number required internationally for a patient subgroup (e.g. 119 HR+/Her2- patients) is reached, the data management and monitoring team will instruct all participating sites to stop the recruitment of patients belonging to this specific subgroup. This monitoring process will prevent any excess accruals and ensure that only the required accrual numbers for each cancer subgroup by subtype are achieved. In case a patient signed the informed consent form on the same day when the required patient number was reached, the patient can still participate in the study.

The competitive recruitment will be closed when the respective numbers of patients to enrol from each cancer subgroup by subtype are achieved as shown in the following tables:

Cancer subgroup by subtype	percentage of patients	fully evaluable patients	patients to be enrolled (based on DO rate)
HR+/HER2-	20%	107	119
HR-/HER2-	40%	213	237
HR+/HER2+	20%	107	119
HR-/HER2+	20%	107	119

Required patients in each cancer subgroup by subtype for Phase I (target of 80 fully evaluable patients):

Cancer subgroup by subtype	percentage of patients	fully evaluable patients	patients to be enrolled (based on DO rate)
HR+/HER2-	20%	16	18
HR-/HER2-	40%	32	36
HR+/HER2+	20%	16	18
HR-/HER2+	20%	16	18

Analysis will be performed when minimum number of patients is reached for every cancer subgroup by subtype. Recruitment per cancer subgroup by subtype will not be closed until the target numbers of patients are reached for phase 2.

Required patients for Phase II (target: 454 fully evaluable patients):

Cancer subgroup by subtype	percentage of patients	fully evaluable patients	patients to be enrolled (based on DO rate)
HR+/HER2-	20%	91	101
HR-/HER2-	40%	181	201
HR+/HER2+	20%	91	101
HR-/HER2+	20%	91	101

Recruitment will be stopped when required numbers of patients per subgroup by subtype are reached.

The study aims to enrol approximately 594 patients (in combination with the BREVITY-02 study in Germany).

The target patient enrolment by country is as follows: 45 patients in 5-6 centres in the US, 82 patients in 5-6 centres in Canada, 145 patients in 12-15 centres in Italy, 100 patients in up to 15 centres in France, 100 patients in up to 15 centres in Spain, in addition to 150 patients in up to 15 German centres (in accordance with the protocol of the BREVITY-02 study RnaDx -BRV-BC-02). These target patient accrual numbers may vary (can be increased or decreased) depending on the patient accrual speed in each participating country and patient accrual will be stopped when the required total number of fully evaluable patients will be achieved globally.

The protocol of the BREVITY-02 study in Germany is in full concordance with the protocol of the international BREVITY study. All data from BREVITY-02 will be amalgamated with international BREVITY and analysed as a unified data set.

4.3 Anticipated enrolment period

The enrolment period is estimated to be 18 months or until all the required patients are enrolled.

4.4 Duration of patient participation

Patient participation will consist of a first stage that requires 2 biopsies during treatment and a second stage of clinical follow-up. The first stage will be for the period of SoC neoadjuvant chemotherapy +/- antibodies +/- other drugs until surgery (~ 5 months duration), while the follow-up duration will be 60 months from the date of enrolment. Patient follow up will be carried out according to guidelines *e.g.* every 3-6 months for the first 3 years then every 6 months for the years 3-5.

4.5 BREVITY study (in combination with BREVITY-02 in Germany) anticipated timelines for phase 1 and phase 2

Trial Phase	Targeted patient accrual number achieved	Surgery results available	3 years survival results available	5 years survival results available
Phase 1 (Training set)	October 2021	April 2022	October 2024	October 2026
Phase 2 (Validation set)	April 2022	October 2022	April 2025	April 2027

4.6 Withdrawal criteria and procedures

Subjects may withdraw their consent for the study and have their specimens destroyed. Subjects may withdraw consent at any time by contacting their clinician without giving a reason. If the patient withdraws consent, the clinician will notify the CRO. Subjects who withdraw their consent will be replaced. Data obtained from specimens prior to receiving a request for destruction will not be deleted. In addition, any analyses in progress at the time of request for destruction or already performed prior to the request being received will continue to be used as part of the overall study data and results. No new analyses will be generated after the request is received. In the event that the medical records for the patient are no longer available (*e.g.* if the investigator is no longer required by regulatory authorities or institutions SOPs to retain the study records) or the specimens have been completely anonymized, there will no longer be a link between the subject's personal information and their specimens. In this situation, the request for specimen destruction cannot be processed.

4.7 Estimation of study completion

It is estimated that the full completion of the study will take up to 7 1/2 years (18-24 months of active patient engagement followed by 5 years of follow-up and ~6 months of data analysis).

5. STUDY DESIGN

5.1 Type of study design

This is a prospective diagnostic performance two-phase multicentre study to be conducted at selected cancer institutions internationally in Europe, the United States and Canada. The study will be performed in neoadjuvant primary breast cancer patients by taking tumour core needle biopsies during treatment. If only one neoadjuvant treatment regimen is planned for a given patient, then biopsies will be taken from the patient at T1 (35 +/- 4 days) as well as (if possible) at a second timepoint T2 at day 55 +/- 5 days. If the treatment is sequential for example 3-4 cycles of regimen A followed by 3-4 cycles of regimen B, then core biopsies are taken at time T1 (35 +/- 4 days) after the initiation of regimen A as well as at time T2 (2 to 3 weeks) after the initiation of regimen B (please follow the specific biopsy collection time points shown in Table 1).

The biopsies will be used to perform RDA analysis and generate RDI scores. The technical success

rate of RDA testing including biopsy material adequacy will be evaluated at Rna Diagnostics' laboratory on a regular basis in order to ensure that the expected number of evaluable samples will be achieved. The inadequacy rate of the biopsies is expected to not exceed 5% in BREVITY. The inadequacy rate will be assessed, during BREVITY at mid recruitment of phase 1 (after 40 patients accrued) with a reassessment at the end of phase 1 (after accrual of 80 patients).

The RDI scores will NOT be communicated to the investigators in order to avoid influencing their treatment decision making. To avoid bias, on the one hand, the pathologist and investigator will be blinded to the RDI scores and on the other the RDA operator will be blinded to the pathology results until the RDI scores are produced and the statistical analysis is performed. Throughout the program, patients will receive standard of care neoadjuvant chemotherapy treatments including taxanes, anthracyclines or other targeted drugs and drug combinations as prescribed based on the investigators' / clinicians' choice. Adjuvant therapies (e.g. radiotherapy, hormonal treatment … etc.) may be prescribed to patients according to standard of care and independently of the RDI score results. RNA disruption will be measured by the RNA Disruption Assay™ (RDA™) from each biopsy specimen separately. The RDA testing will be performed by trained operators at a centralised testing facility (Rna Diagnostics, Sudbury, Ontario, Canada). Clinical and patient outcome data (surgery and 5 year survival data) will be sent to Rna Diagnostics for correlations to the RDI scores and further statistical analysis. The study consists of a phase 1 (training set) to determine the test cut-offs between the response zones using pCR outcomes, and a validation phase 2 to demonstrate the performance characteristics of the RDA test using both correlation to pCR and correlation to DFS as endpoints. Patients from phase 1 will be excluded from the analysis of the validation phase.

5.2 Schematic of BREVITY study design

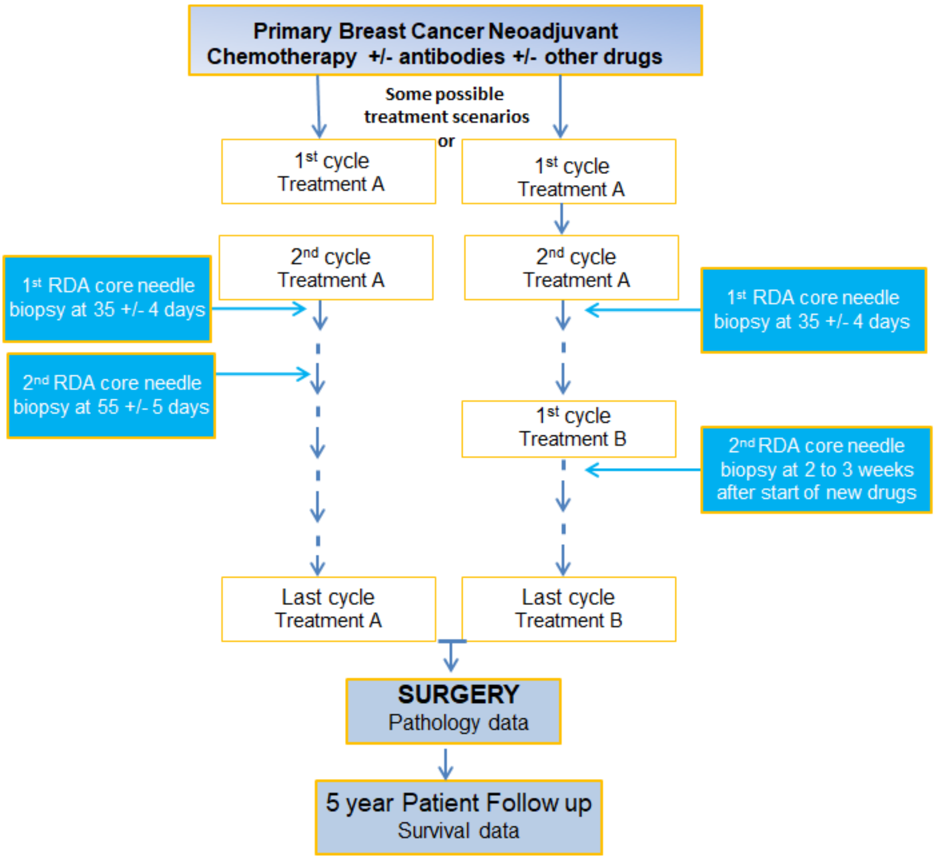

Figure 1: RDA BREVITY study design (5 year follow ups will be carried out according to guidelines).

5.3 Timing of sample collection

After giving informed consent, an eligible patient will become a participant with an assigned coded number. Tumour RDI score will be evaluated by obtaining two core needle biopsy specimens (to account for tumour heterogeneity) at each collection time point.
Table 1 below shows the collection time points to use for performing the biopsies in each case.

Table 1. Biopsy Collection Time Points		
Biopsy	*1st core needle biopsy for RDA (2 specimens)*	*2nd core needle biopsy for RDA (2 specimens)*
Biopsy timing by type of drug schedule	*35 days +/- 4 days*	***If no change of drugs:***<br>*55 days +/- 5 days*<br><br>***If there is a change of drugs:***<br>*2 to 3 weeks after start of new drugs as follows depending on drug regimen:*<br>• *3-weekly: at 16 days +/- 2 days*<br>• *Bi-weekly: at day of 2nd dose preferably before drug admin.*<br>• *Weekly: at day of 4th dose preferably before drug admin.*

5.4 Study parameters and enrolment procedure

The information summarized in the parameter list below will be collected on all the patients enrolled and will be communicated to Rna Diagnostics after the RDI scores are released. The CRO will send to Rna Diagnostics the patient clinical and surgery outcome data when the target 80of fully evaluable patients for the training set has been reached internationally. Correlation to pCR will be performed in phase 2 (validation set) as soon as surgery results are available for the target 454fully evaluable patients. Also, long-term follow up data will be communicated to Rna Diagnostics to allow performing survival analysis both at 3 years and at 5 years.

Table 2. Study Parameters	
	Parameters
**Demographics**	▪ Age<br>▪ Weight and height<br>▪ Menopausal status (not mandatory, only if available)
**Primary Disease**<br>Initial diagnosis of primary disease:	▪ Tumour size<br>▪ T stage: T1, T2, T3<br>▪ Stage: I, IIA, IIB, IIIA, IIIB, IIIC<br>▪ Grade: 1, 2, 3<br>▪ Ki 67 if available (not mandatory)<br>▪ Receptor status: ER, PR, Her2, TNBC

Neoadjuvant therapy:	▪ Drugs administered (taxanes, anthracyclines and/or other drugs incl. targeted therapies…)<br>▪ Dosage of drugs and duration with start and end dates
Biopsies for RDA testing	▪ Biopsy material adequacy<br>▪ RDI scores
Surgery and pathology:	▪ Date of surgery<br>▪ Type of surgery<br>Residual cancer burden (size of residual tumour if any, number of positive lymph nodes / total lymph nodes dissected, cellularity of residual tumour and other measurements required for RCB calculation based on the MD Anderson RCB calculator guidelines http://www3.mdanderson.org/app/medcalc/index.cfm?pagename=jsconvert3).<br>▪ Receptor status in residual tumour (if available)
Post-Surgery and throughout enrollment:	▪ Types of adjuvant therapies (e.g. tamoxifen, other drugs, radiotherapy … etc.) with doses and duration with start and end dates<br>▪ 3 year and 5 year disease free survival<br>▪ Overall survival<br>▪ Type of 1st relapse (local/distant), location of lesions, date of detection.<br>▪ A primary serious adverse event that has caused death other than due to cancer progression

5.4.1 Screening

The site investigator or site coordinator from each cancer centre will screen referral patients for eligibility to participate in the study. The site investigator will describe the study to eligible patients during initial consultation and ask if the patient is willing to consent to participate in the study. Recruitment information (please see patient information leaflet and consent form) will be given to the potential participant. If the patient is willing to participate, the site coordinator will make an appointment for the baseline visit with the patient preferably before the 1st cycle of neoadjuvant chemotherapy but no later than the collection timepoint of the 1st RDA biopsy which is the study procedure. A screening failure is a patient who was recruited but was found to not meet one or more criteria required for participation in BREVITY before the 1st biopsy collection timepoint.

5.4.2 Consenting and registration

All subjects who volunteer to participate in the study will be required to provide their written informed consent before any study procedure starts (before 1st RDA biopsy collection). The patient consent form will be provided with the protocol for review and approval by the IRB/IEC at each institution in advance of use. Informed consent will be obtained from each volunteer according to applicable regulatory and legal requirements before she donates her biopsy specimens. The consent must be signed and personally dated by the subject and by the person conducting the consent discussions. The original signed consent form will be retained at the collection site as part of the study records. A copy of the signed Informed Consent Form should be provided to the subject for her to keep.

5.4.3 Sample and data collection

Specimens will be collected by core needle biopsy according to the institution's standardized procedures. Collection supplies that include RDA biopsy collection tubes with fixative and labels

will be provided by Rna Diagnostics. Detailed instructions on sample handling, processing and shipping is provided in Appendix B.
Two core needle biopsy specimens should be collected from each consenting subject, at each collection time point. If only one neoadjuvant treatment regimen is planned for a given patient, then biopsies will be taken from the patient at T1 (35 +/- 4 days) as well as (if possible) at a second timepoint T2 at day 55 +/- 5 days. If the treatment is sequential for example 3-4 cycles of regimen A followed by 3-4 cycles of regimen B, then core biopsies are taken at time T1 (35 +/- 4 days) after the initiation of regimen A as well as at time T2 (2 to 3 weeks) after the initiation of regimen B (specific time points of biopsy collection for each type of drug schedule are shown in table 1).

The following information will be recorded for each biopsy sample collected:

- Date and time of the sample collection;
- Neoadjuvant chemotherapy type and cycle;
- Date and time of the last dose of chemotherapy before taking the sample.

5.4.4 Follow-up

Patients will be followed-up for a period of 60 months from the date of enrolment. Patient drop out and loss to follow up can occur after the beginning of neoadjuvant therapy. The following actions must be taken if a participant doesn't return to the clinic for a required study visit:

- The site will make effort to contact the patient and schedule a new appointment;
- The patient will be informed about the importance of a regular consultation at the site if she wants to continue participating in the study;
- After 3 attempts without success, a patient will be regarded as a "loss to follow-up";
- The contact attempts (by phone or post) will be documented in the patient file.

6. SAFETY ASSESSMENT

6.1 Safety assessments considerations

Patients for this trial will be treated according to local SoC. Local safety assessments for SoC has to be followed by the Investigators.
Additional tumour core-needle-biopsies must be taken during neoadjuvant chemotherapy. Core needle biopsy procedures are considered safe and well established. Bleeding, haematoma and infection of the biopsied site are possible adverse events related to the biopsy procedure.
In addition, published study reports indicate that preoperative core needle biopsies do not influence local cancer recurrence rate and patient survival [11, 12]. Therefore, an increase of risk of cancer recurrence because of the core needle biopsies in BREVITY is not expected. Any related incident or risk identified as potentially due to the additional biopsies will nevertheless be evaluated and reported as part of the SAE reporting process in place for the full duration of the BREVITY study including 5 years of patient clinical follow-up (detailed in section 6.2.).

The following safety parameters should be taken-into account prior to the biopsy procedure to reduce patient's risk during participation in this trial as far as possible.

6.1.1 Physical examination

The area to be biopsied needs to be inspected for skin infections. In case of an infection, the biopsy should not be taken.

6.1.2 Clinical safety laboratory assessment

The following laboratory results may be assessed, at the recommendation of the investigator, before the core-needle-biopsy procedure takes place. It is expected that these laboratory results

would be available before the biopsy and taken as part of the safety assessment for SoC neoadjuvant treatment.
The core-needle-biopsy procedure should not be done, if the following ranges are exceeded:

- INR or Quick ≤ CTC Grade II
- Thrombocytopenia ≤ CTC Grade III
- Neutropenia ≤ CTC Grade III

6.2 Reporting of serious adverse events

A serious adverse event (SAE) is any untoward occurrence in a clinical investigation/performance evaluation which is subject to approval that leads, could lead or could have led directly or indirectly to death or serious deterioration of general health of a test person, an end user or any other person. It is irrelevant whether the event has been caused by the medical device or not.

Serious adverse events (SAEs) are events that lead to:

- Death or
- Serious adverse health effects, that result in:
  - life-threatening situations;
  - permanent disability or invalidity;
  - hospitalisation or prolongation of hospitalisation;
  - medical or surgical intervention to prevent permanent impairment of body structures or body functions;
  - damage of fetus, fetal loss, congenital malformation or birth defect.

Planned inpatient treatments of pre-existing conditions or scheduled measures according to the protocol are not SAEs as long as there is no serious deterioration of the patients' physical condition.
The investigator will be asked to share serious adverse events for the sponsor's records according to local laws and regulatory requirements. The investigator is obliged to report <u>any serious adverse event</u> that has a causal relationship with the study procedure (the RDA biopsies) or where a causal relationship is reasonably possible within 24h after becoming aware of the SAE. The SAE should be reported by completing the corresponding page in the eCRF.

Follow-up of SAEs: after the initial SAE report, the investigator is required to proactively follow each participant at subsequent visits/contacts. All reported SAEs will be followed until resolution, stabilization, the event is otherwise explained, or the participant is lost to follow-up.

If during the conduct of the clinical investigation/performance evaluation circumstances arise that could adversely affect the safety of a test person, end user or third person, the sponsor will take, without delay, necessary measures to protect them against direct or indirect danger. The sponsor will inform the concerned competent authorities and ethics committees about these new circumstances immediately.

6.3 Risk – benefit assessment and COVID-19 related risk mitigation measures

In addition to possible side effects during the biopsy procedure, potential biosafety related risks include accidental events *e.g.* core needle biopsy related injury or infection that could affect the patient or the clinical personnel involved in BREVITY. A full list of potential risks and a detailed assessment and description of risk mitigation measures is provided as an attachment (RDx_RMT_01). A new risk that has been identified pertains to the conduct of the international BREVITY trial during the COVID-19 pandemic. On the one hand, the BREVITY trial doesn't offer a direct benefit to patients participating in the trial; therefore, any risk related to COVID-19 could contribute to a further decrease of the benefit / risk ratio. On the other hand, BREVITY aims to validate RDA as a drug efficacy predictive tool that can possibly add great societal value in the

future. The validation of the RDA test could help to optimize patients' neoadjuvant therapy, enhance patients' survival and reduce the toxicity of ineffective drugs. With the potential future societal benefit of BREVITY as a validation trial, the risk / benefit ratio in its broader definition is significantly improved and is a compelling reason to complete the trial.
Moreover, the incremental risk from the conduct of BREVITY during the COVID pandemic will be mitigated further by implementing several mitigating measures. With appropriate risk mitigation measures in place as described in the risk assessment and management attachment [13], the risk to patients participating in BREVITY during the COVID-19 pandemic is expected to be lowered to an acceptable level, in situations where all governmental policies permit it and ethics committee approvals have been obtained. The risk mitigation approach that the BREVITY team will implement will take in consideration that COVID-19 pandemic is an unprecedented situation and will be adjusted in time as needed. By recognizing that the situation can be fast changing, all the BREVITY team (Sponsor, PIs, medical monitors, CROs, steering committee, etc.) will perform ongoing monitoring and re-assessments of all related risks to ensure that precautionary measures are continuously re-adjusted to remain as effective and as appropriate as possible.

7. INFORMATION/ PROCEDURES ON SAMPLES

7.1 Sample processing

Biopsies will be preserved in a fixative then sent to Rna Diagnostics for further processing. When received at Rna Diagnostics, RNA will be extracted from the tumour biopsies and analysed for its quality in a bio-analyzer that produces RNA quality electronic files (.xad files). The .xad electronic files from the bioanalyzer will be used to perform RDA testing with RDA's proprietary algorithm. Rna Diagnostics will perform the RDA within 2 weeks from the reception of the biopsy specimens. The remaining RNA isolated from the tumour biopsies will be stored in the RNA diagnostics laboratory in a -20ºC or -80ºC freezer for further research – this could be for up to 10 years before being destroyed.
FFPE blocks of biopsy taken at pre-therapy for diagnosis saved in the participating centre facility may also be requested by Rna Diagnostics for use in further analysis. This will be contingent upon ensuring that sufficient material will remain in the participating centre for further diagnostics.
Sample processing at Rna Diagnostics' laboratory is described in Appendix B.

7.2 Storage and transport of samples

The biopsy specimens collected in tubes with RNAlater fixative (Catalog # AM7021 or AM7024, Thermo Fisher Scientific (https://assets.thermofisher.com/TFS-Assets/LSG/manuals/7020M.pdf) – manufactured and sold under ISO13485 and ISO9001) will be stored at room (ambient) temperature overnight and then stored in a freezer at the site for a variable period of time (1 to 90 days) until transport to the Rna Diagnostics laboratory. The Rna Diagnostics laboratory should be notified of the shipment of the biopsies. The costs for sending the samples will be covered by Rna Diagnostics.

At the participating site, the biopsy specimens are stored in RNAlater, an RNA stabilization solution. This is a tissue storage reagent that enters the tissue, stabilizes the sample and protects the cellular RNA. According to the product information sheet for RNAlater, samples can be stored at ≤ 37°C for a day, at ≤ 25°C for a week, at 4°C for one month, and indefinitely at -20°C or -80°C. RNA Diagnostic's stability studies have shown that the RNA disruption index (RDI) of samples stored in RNAlater for up to 4 days at room temperature is unaffected. More specifically, an overall exposure to 23°C for up to 96 hours or an exposure at 37°C for up to 24 hours, has been shown to have no impact on the RDA testing results. Therefore, the goal of the freezer and shipment temperature monitoring and management is to ensure that the biopsies do not sit at room temperature for more than 4 days or at 37°C for more than 1 day from the time they are collected

and stored in the freezer at the site to the time they are received at Rna Diagnostics' laboratory to perform the RDA testing.
Collection and shipment of all biopsies will be documented in a tracking list at each site. A sponsor approved temperature logger must track the storage temperature of the biopsies during transport.
Please refer to **Appendix B** for detailed core biopsy collection and storage guidelines.

1. Cold chain monitoring during storage at the sites:

The biopsy specimens should be stored such that the temperature is -10°C or less. This should be done in the site's -20°C freezer but in the absence of a -20°C freezer, a -80°C freezer is acceptable. Both -20°C and -80°C freezers will be acceptable if they are, at a minimum, equipped with a display of the internal temperature of the freezer. In addition, any one or more of the following measures should be in place to ensure the stability of the biopsy specimens:

a) The freezer will be checked once daily on business days to ensure that the temperature is maintained at -10°C or lower; or
b) The freezer will be equipped with a local audible alarm system such that, in the event of an unexpected warming of the freezer, the alarm system is triggered; or
c) The freezer will be monitored electronically, where the freezer's temperature is measured and logged automatically, and the freezer and the freezer logs are manually inspected on a twice-weekly basis; or
d) The freezer will be equipped with a temperature alarm system, which will be connected to the cell phone of staff who will be alerted by call or text message in the event of an unexpected warming of the freezer that triggers an alarm.

During storage in the freezer at the site, in case of any unexpected warming event due to freezer malfunction or mishandling (e.g. frequent freezer door opening during daily use, or accidentally leaving the freezer door ajar), whenever the temperature exceeds -10°C (a "temperature excursion"), it is required that the site informs the CRO Stiris Research of the event. Temperature excursions during storage in the freezer and their estimated durations together with a list of biopsies affected will be communicated to Stiris Research, and also by Stiris Research to the Rna Diagnostics laboratory.

2. Cold chain management during shipment of biopsy specimens:

a) Shipping day: Tumour biopsies can be stored at the site per the instructions above for up to 90 days. The biopsy samples will be preferably shipped on Mondays in order to mitigate any risk of delay due to weekends. Biopsies shipped by Fedex have usually arrived at Rna Diagnostics' laboratory in Canada within 2-3 days. Shipments rarely took more than 4 days to arrive.
b) Shipment box and cold packs: Each biopsy shipment Styrofoam box will contain 4 pre-frozen cold packs together with the biopsies and other materials described in the "biopsy specimen packaging instructions manual". The 4 cold packs will be pre-frozen by placing them in the freezer at least 24 hours before and until shipment. The 4 cold packs are expected to maintain the temperature below 23°C for up to 4 days. Given that the shipment duration rarely exceeded 4 days, combined with the fact that the samples are stable for up to 4 days even if the temperature does reach 23°C, the 4 cold packs will suffice in this case.
c) Temperature control: One iMINI USB pdf temperature logger (Cryopak, Canada) or sponsor approved equivalent will be added inside the Styrofoam box on top of the Cryobox containing the biopsies. The approved temperature loggers are programmed to measure the temperature of the container at least every 30 min from the time of activation. This will allow a recording of the internal temperature of the Styrofoam box for a time period of at least 55 days, which covers the entire duration of the biopsy shipments. The temperature loggers are also equipped with two temperature limits (23°C and 28°C). If the shipment temperature

exceeds one or both limits, one or both alarm LEDs will be activated. The temperature loggers are CE-marked and NIST-traceable.

As soon as the biopsies will be received at the Rna Diagnostics laboratory, the temperature log will be downloaded and filed during opening of the biopsy shipment Styrofoam box. Any event where (i) the temperature has exceeded 23°C, and the lower alarm is triggered, and/or (ii) the shipment duration exceeded 4 days, the temperature log will be inspected. If required, Rna Diagnostics will analyze and investigate the situation further to determine if temperature excursions have occurred. Samples will be excluded if they A) exceed 37°C for any period of time, B) are at a temperature of greater than 30°C for more than 18 cumulative hours, C) are at a temperature of greater than 25°C for more than 48 cumulative hours or D) are at a temperature greater than 20°C for more than 96 cumulative hours.

A document listing biopsy IDs, related temperature excursions, if any, with their estimated durations (where applicable) during both the site storage and the shipping stage will be kept and updated on a routine basis at the Rna Diagnostics laboratory. In the event of temperature excursions during the freezing and/or shipment, the overall temperature exposure will be investigated by the scientific and clinical team at Rna Diagnostics to evaluate its validity for inclusion in the BREVITY trial data analysis. If an impact of a temperature breach on the RDA result cannot be ruled out, the corresponding biopsy/biopsies will be excluded from the analysis.

7.3 Facilities to be used and contact details

For contact details please see the protocol cover page.

8. IDENTIFICATION OF SOURCE DATA/ DATA TO BE ENTERED ON CRFS

Data will be recorded on electronic Case Report Forms CRFs using the Electronic Data Capture EDC system provided by the international management CRO Advanced Medical Services GmbH (AMS). CRFs may be verified against all original records by the study monitor. CRFs and all original data should be readily available for review during scheduled monitoring visits.

CRF completion instructions will be provided for each site by the local CRO Stiris Research and any questions about the appropriate location on the CRF for recording specific information should be directed to Stiris Research.

In order to minimize potential bias of the study, the following precautions will be implemented:

- Biopsy specimens' information will be coded so that operators responsible for testing patient specimens with RDA assay and interpreting test results will not have any prior knowledge of clinical and patient outcome data until the RDI scores are released.
- Investigators / clinicians should stay blinded to the RDI scores during the patient treatment in order to avoid acting on the RDI information.
- Pathologists responsible for the interpretation of post neoadjuvant specimens should also be kept blinded to the RDI scores until the pathology report is released.

CRFs will be used to collect patient and clinical data corresponding to the parameters listed above in the Table 2.

9. STATISTICAL CONSIDERATIONS

9.1 RDI scores

To account for intra-tumoural heterogeneity, 2 RDI scores will be generated from two separate tumour specimens at each biopsy collection time. The patient's official RDI score will be determined as follows:

- If only 1 sample generates an evaluable score, this will be the official RDI score for the patient;

- If 2 or more evaluable scores are generated for a given patient, possibly at different biopsy collection timepoints, the official RDI score will be the highest RDI score;
- If no samples give an evaluable score, this would be a case of missing value that would be handled as described below in section 9. 7. “Handling of missing values”.

9.2 Rationale of statistical design

The statistical design of the study has a twofold primary objective:

1. Select a suitable parametrization for the diagnostic test in terms of two cut-offs in RDI parameter space (Phase 1).
2. Evaluate performance characteristics of the diagnostic test for selected cut-offs (Phase 2).

The two cut-offs divide the RDI parameter space on which the test operates into three zones. Zone 1 is associated with lower pCR prevalence. It is to be defined by cut-off c_1, which will be selected to optimize the balance between a significant percentage of patients falling into Zone 1 to enable good clinical utility, while also maintaining a high negative predictive value (NPV). As a result, patients tested as “zone 1” will have a low probability of reaching pCR with the current neoadjuvant treatment and may benefit from a change of treatment. This recommendation represents the main clinical utility of the diagnostic test.

A second cut-off (c_2), defining zone 3, will be chosen to optimize the positive predictive value (PPV). It is hypothesized that for certain subgroups, such as e.g. HER2+ patients, a classification as “zone 3” may provide additional clinical utility in predicting a positive outcome of neoadjuvant therapy in terms of pathological complete response (pCR).

The twofold primary objective is realized by a corresponding two-stage design, as shown in Figure 2 below. In a first step, the cut-offs are calibrated on a training set of patients with respect to positive and negative predictive values. The clinical outcome under consideration is pCR. In a second step, the diagnostic test’s predictive values resulting from the calibration on the training set are evaluated on an independent validation set. Evaluating the test on an independent validation set eliminates the chance of bias due to overfitting when establishing the performance characteristics. As described in the statistical plan[14] - the performance claims to be validated in BREVITY are diagnostic sensitivity, diagnostic specificity, and diagnostic accuracy.

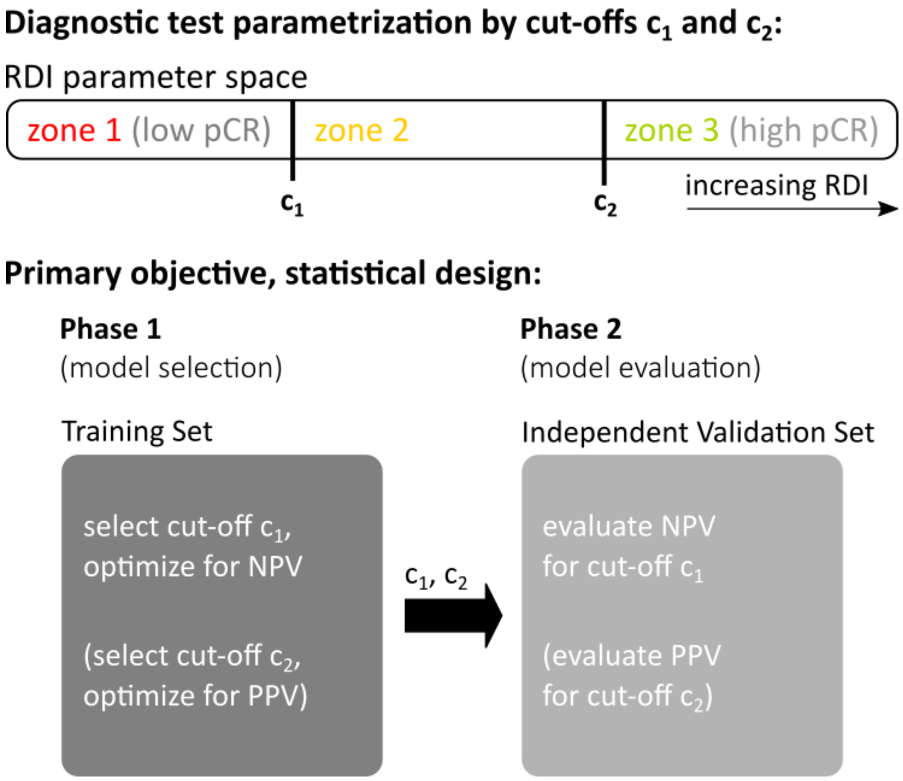

Figure 2: Parametrization and two-stage validation strategy for the RDI diagnostic test.

Note that, in principle, the test's bio-chemical mechanism is identical regardless of therapy or clinical cancer subtype. On the other hand, the NPV is a function of pCR prevalence in the population, and thus depends on both. However, prior study data [1] indicate that optimal ranges for a cut-off with respect to NPV (i.e. maintaining a high NPV while having a significant percentage of patients falling into Zone 1) will vary only very slowly with pCR prevalence. In particular, optimal cut-offs were seen to be invariant across subgroups. As a result, calibration and validation of cut-offs will be pooled across subgroups in this study.
A dedicated secondary objective investigates NPV absolute values within the different cancer type subgroups as supportive analysis. In addition, a test for association of cancer type subgroups and test results will be carried out to verify the assumptions underlying the pooling strategy.
Due to the high clinical relevance of cancer type subgroups, a number of subgroup analyses with regard to clinical outcome (DFS, pCR) are planned as secondary supportive objectives and are considered to be necessary in order to further explore the test characteristics. To address concerns regarding the robustness of subgroup analyses, the latter will focus on reporting and interpreting confidence intervals of effect size. This way, uncertainty pertaining to observed effects is quantified and should be taken into consideration when interpreting the results.

9.3 Primary analysis

In phase 1, the full training set data will be used to estimate and visualize operating characteristics as functions of RDI. These include NPV, PPV, sensitivity, specificity, as well as related concepts. Based on these provisional analyses, suitable cut-offs will be determined that optimize a balance of clinical utility (i.e. percentage of patients) with NPV of zone 1 and PPV of zone 3 by expert adjudication. The expert group tasked with adjudication will consist of several scientists and physicians with extensive experience in test development and validation along with a biostatistician.
In phase 2, the analysis will proceed by interval-estimation of performance characteristics on an independent validation set of patients, given the cut-offs selected on the training set in phase 1.

The primary target of validation will be the NPV of zone 1 which corresponds to the test's main clinical purpose. The PPV of zone 3 will be established on the validation set for selected subgroups in a dedicated secondary objective.
The primary objective for validation is the estimation of the NPV associated with zone 1 by a 95% confidence interval where the validation set is powered to yield a precision of 5%, as defined in the following sections. Figure 2 summarizes the primary analysis.

9.4 Determination of sample size

Phase 1:
Based on proprietary analyses of data from previous studies [1] that measured RDI, it is hypothesized that good candidates for cut-offs c_1 and c_2 are the 25th and 75th percentile of the RDI distribution across patients. With regard to c_1, the data shows a very high NPV value below the 30th quantile which changes only moderately as a function of RDI. In particular, the estimated NPV was above 95% in in the MA.22 study [1] in this part of parameter space, with a pooled approximate pCR rate of 10% in the corresponding patient collective.
Based on these preliminary considerations, phase 1 will be powered to estimate the 25th RDI percentile as a candidate for cut-off c_1 on the training set with a precision E of 9.5%, which was deemed acceptable for the purpose of expert adjudication.
E is defined in this context as the maximum tolerated error in estimating the q-percentile ξ_q in terms of a 95% confidence interval in units of q, given by $[\xi_{q-E}, \xi_{q+E}]$. If x_k denotes the kth sample from an ordered set of size n, the k realizing the event $[x_k \leq \xi_q]$ is binomially distributed with parameters q and n. As a result, k/n is approximately normally distributed with expectation q and variance $\frac{q(1-q)}{n}$.
Approximate 95% confidence bounds for q follow as $q \pm z_{0.975}\sqrt{\frac{q(1-q)}{n}}$, where z is a percentile from the standard normal distribution. Consequently, following Chow et al. [15],

$$n = \frac{z_{0.975}^2 q(1-q)}{E^2}.$$

For q = 0.25 and E = 0.095, a sample size of n = 80 evaluable is thus obtained for the training set of phase 1. Accounting for 10% analysis dropouts, a lower bound for the phase 1 sample size is89. To comply with stratification requirements (see sec. 4), n = 90 is set to be enrolled for phase 1.

Phase 2:
The objective in phase 2 is to establish unbiased diagnostic test performance on the validation set in terms of the NPV for zone 1. The sample size n for the validation set is chosen to yield a 95% confidence interval estimate for the NPV with a precision of 5.25%. To this end, consider the following contingency table representing observed frequencies for a sample obtained by the validation set:

	**test +** (zone 2, 3)	**test –** (zone 1)	
**pCR** = 1	s_1	s_0	n_1
**pCR** = 0	r_1	r_0	n_0
	n_+	n_-	$\boldsymbol{n}$

A point estimator for the true NPV is calculated on the sample by $\frac{r_0}{n_-}$ and will be asymptotically normally distributed with expected value given by the true NPV and variance by $\frac{NPV(1-NPV)}{n_-}$.

Assuming again an optimal cut-off for c_1 will be given by the 25th percentile of the RDI distribution across patients, we have $n = \frac{n_-}{0.25}$ where n_- is computed as in phase 1 for CI precision E of 5.25% following Chow et al. [15]. Hence the sample size n is calculated by

$$n = \frac{z_{0.975}^2 NPV(1 - NPV)}{0.25E^2}$$

For the sample size calculation, a point estimator for the true NPV in the validation set is derived at 91.1% based on the following considerations. According to Cortazar et al. [10] and interviews with investigators, the following breakdown of cancer subtypes and pCR rates is expected for neoadjuvant therapy:

subtype	percentage of patients	pCR rate
HR+/HER2-	20%	16.2%
HR-/HER2-	40%	43.3%
HR+/HER2+	20%	35.1%
HR-/HER2+	20%	62.4%

This would correspond to an expected pooled pCR rate of 40.1% in the validation set. Using interpolated joint estimates of sensitivity and specificity from the MA.22 study, the estimated NPV at the pooled pCR rate obtains at 91.1%.

With NPV assumed to be 91.1% in the validation set and choosing precision E = 0.0525, a sample size for phase 2 is thus obtained as n = 452 evaluable. Accounting for 10% dropouts, a lower bound for the phase 2 sample size is 502. To comply with stratification requirements (see sec. 4), n = 504 is set to be enrolled for phase 2.

9.5 Analysis populations

**ITT (intent to treat)**	The primary analysis population is the ITT collective, consisting of all patients that are successfully screened and allocated to neoadjuvant therapy.
**PP (per protocol)**	For sensitivity analyses, a per-protocol collective is defined as a subset of ITT, consisting of all patients that complete neoadjuvant treatment as planned.

9.6 Statistical methods

Objectives	Statistical methods	Analysis population	Data
**Primary objective** Phase 1	Determination of cut-offs c_1, c_2 on training set by expert adjudication.	PP	training set
**Primary objective** Phase 2	Interval estimation of “zone 1” NPV on validation set by 95% confidence interval (Wilson Score interval).	ITT, PP	validation set

**Secondary Objectives**			

i. Interval estimation of NPV in main cancer type subgroups:<br>HR-/HER2-,<br>HR-/HER2+,<br>HR+/HER2+,<br>HR+/HER2-	(1-α)100% confidence intervals (Wilson Score interval), Bonferroni correction of local α to yield a global type I error rate of 0.05 for this objective.	ITT, PP	validation set
ii. Interval estimate of PPV in HER2+ subgroup	95% confidence interval (Wilson Score interval) in subgroup of all HER2+ patients.	ITT, PP	validation set
iii. Assess pCR rate for zones 1-3 in cancer type subgroups:<br>all patients,<br>HR-/HER2-,<br>HR-/HER2+,<br>HR+/HER2+,<br>HR+/HER2-,<br>all HER2+,<br>all HER2+ and HR-/HER2- combined	For each indicated cancer type subgroup, the following analyses will be performed:<br>• Calculation of raw p-values for two-sided Fisher's exact test,<br>• corresponding exact 95% CI for the odds ratio<br>Each test is computed for each of the following comparisons:<br>1. H0: equal pCR rates, zone 1 vs. zones 2,3<br>2. H0: equal pCR rates, zone 1 vs. zone 3<br>3. H0: equal pCR rates, zone 3 vs. zones 1,2<br>Additional simultaneous confidence intervals for odd ratios will be provided across subtypes and comparisons for global α=0.05.	ITT, PP	validation set
iv. Assess DFS for zones 1-3 in cancer type subgroups:<br>all patients,<br>HR-/HER2-,<br>HR+/HER2+,<br>HR+/HER2-,<br>HR-/HER2+,<br>HER2+,<br>HR-/HER2- and HER2+,<br>HR-/HER2+ and Luminal B	For each indicated cancer type subgroup, the following analyses will be performed:<br>• Calculation of raw p-value for log-rank test,<br>• corresponding hazard ratio with 95% CI,<br>• Kaplan-Meier estimator,<br>• median and 5-year DFS with 95% CI<br>Each test is computed for each of the following comparisons:<br>1. H0: equal DFS, zone 1 vs. zones 2,3<br>2. H0: equal DFS, zone 1 vs. zone 3<br>3. H0: equal DFS, zone 3 vs. zones 1,2<br>Additional simultaneous confidence intervals for hazard ratios will be provided across subtypes and comparisons for global α=0.05.	ITT, PP	validation set
v. Test for association between subgroups and test result	Chi-squared test for difference in zone 1 prevalence between the four groups of main cancer subtypes (HR+/HER2-, HR-/HER2-, HR+/HER2+, HR-/HER2+) among subgroup of patients without pCR.	ITT, PP	validation set

Analyses of single proportions will consist of reporting 95% confidence intervals based on the Wilson-Score method by Newcombe [16].

Tests of proportions will be carried out by Fisher's exact test with corresponding (1- α)100% confidence intervals for the odds ratio [17]. Raw p-values will be reported without reference to significance thresholds.

Survival analyses will consist of log-rank tests with corresponding (1-α)100% CI for the hazard ratios (based on uni-variate Cox regression models [18]). Raw p-values will be reported without reference to significance thresholds. In addition, Kaplan-Meier estimators with confidence bands are reported, as well as median and 5-year DFS with 95% confidence intervals.

For subgroup analyses in secondary objectives, additional simultaneous confidence intervals will be provided based on a Bonferroni correction [19].

Additional sensitivity analyses will explore possible country, center and BC-type effects on the RDI score distribution jointly in a mixed model.

9.7 Handling of missing values

With regard to the two primary parameters, RDI and pCR, every effort will be made to prevent missing data and to acquire the missing values if missingness occurs.
If a clinical sample is not analyzable at RNA level the RDI value is not available and will be missing. A sample is not analyzable if there is insufficient information in the electropherogram, e.g. due to low RNA concentration. This may occur due to a processing error in the RNA fixation or isolation, or due to the biology of the tumour destruction (the RNA concentration decreases with therapy and may become too low to generate an adequate electropherogram). Both mechanisms are not expected to occur commonly in the BREVITY trial at early stages of neoadjuvant therapy.
In addition, to compute the RDI only one of two samples that are routinely taken at a given time point has to be adequate, i.e. one of four samples taken in a context where a change in therapy may be indicated by the test. This further decreases the chances of having missing values due to inadequate samples.
If missing values occur, the primary analysis will proceed on the subset of patients with observable values. Reasons for missingness will be investigated to check for potential bias and will be reported as part of the analysis. It will be tested whether data are not missing completely at random (MCAR). If data are missing at random (MAR), methods for multiple imputation of pCR and RDI may be feasible [17, 20]. At the trial statistician's discretion, robustness of statistical inference has to be established via sensitivity analysis under varying assumptions regarding missingness, including non-ignorable missingness (NI, MNAR). Sensitivity analyses will be carried out by means of an imputation-driven strategy on a case-by-case basis, as recommended by the NRC [21]. Results of sensitivity analyses must be reported as part of the primary analysis resulting from the study data.

10. ADMINISTRATIVE RESPONSIBILITIES

10.1 Good clinical practice

The study will be conducted under the standard "in-vitro diagnostics medical devices – clinical performance studies using specimens from human subjects – Good study practice (ISO 209162019)" and in accordance with ICH-GCP, where applicable. The investigator will be thoroughly familiar with the study requirements as outlined in the protocol. Essential clinical documents will be maintained to demonstrate the validity of the study and the integrity of the data collected. Master files should be established at the beginning of the study, maintained for the duration of the study and retained according to the appropriate regulations.

10.2 Ethical considerations

The study will be conducted in accordance with ethical principles founded in the Declaration of Helsinki (current edition) located at:
http://www.wma.net/en/30publications/10policies/b3/).

An ethics committee will review all appropriate study documentation in order to safeguard the rights, safety and well-being of the patients. The protocol, informed consent, written information given to the patients, annual progress reports and any revisions to these documents will be provided to them by CRO and the sponsor. The study will only be conducted at sites where their full approval has been obtained.
As part of quality system, Rna Diagnostics has an Ethical Conduct SOP. This SOP applies to all Rna diagnostics' management, employees and contractors who conduct Rna Diagnostics business or perform the RDA assay. A copy of this SOP is enclosed with the EC submission documents.
Any proposed future research which is outside the scope of this study and which would involve the use of samples from this study will be subject to a new application for review and approval by the research ethics committee.

10.3 Patient information and informed consent

After the study has been fully explained, written informed consent will be obtained from the patient prior to study participation. The method of obtaining and documenting the informed consent and the contents of the consent will comply with ICH-GCP, the standard "in-vitro diagnostics medical devices – clinical performance studies using specimens from human subjects – Good study practice (ISO 209162019)" where applicable, and all applicable regulatory requirements.

10.4 Patient confidentiality

In order to maintain patient privacy, all CRF, study reports, biopsy sample containers and communications will identify the patient by the assigned patient number. The investigator will grant the monitors access to the patient's original medical records for verification of data gathered on the case report forms and to audit the data collection process. The patient's confidentiality will be maintained and patient names will not be disclosed.

10.5 Steering Committee (SC)

The Steering Committee will consist of the Principal Investigators of the BREVITY participating countries: USA, Canada, Italy, France and Spain (for names and addresses, please refer to page 1), as well as Germany (from BREVITY-02). The SC will be co-chaired by Dr. Foluso Ademuyiwa (Washington University School of Medicine in St Louis, MO, USA) and Dr. Marina Cazzaniga (Hospital Oncologico, ASST di Monza, Monza, Italy).
The role of the SC will be to provide the overall supervision in order to ensure that the trial is conducted in accordance with the principles of Good Study Practice and the relevant regulations. The SC will also provide advice to the investigators on the various aspects of the trial. The SC will meet once a year but meetings may be more frequent if need be.

10.6 Protocol violation (or deviations) and non-compliance

The investigator will conduct the study in compliance with the approved protocol. Changes to the protocol will require written ethics committee approval/favourable opinion prior to implementation, except when the modification is needed to eliminate an immediate hazard(s) to patients. The sponsor and the CRO will submit all protocol modifications to the ethics committee for review in accordance with the governing regulations.
Investigational sites are trained on non-compliance and violations (deviations) of the protocol that might potentially occur during study conduct in order to prevent non-compliance. Non-compliance and violations (deviations) of the protocol can be detected in different ways, e.g.:

- Detection by Data Management by applying edit checks to the data base;
- Investigator proactively notifies sponsor personnel (clinical monitors, PM) of violations of the protocol that have occurred at his investigational site;
- Detection during remote or on-site monitoring visits by clinical monitors;

- Detection during audits or regulatory inspections.

All non-compliance and violations (deviations) of the protocol will be documented and categorized for example as follows: source data, ICF, in- and exclusion criteria, SAE reporting, endpoints (tumour sampling, assessment and evaluability), unblinding of patient identity.
Non-compliance or violations (deviations) of the protocol assessed as serious breaches are reported to the ECs (leading EC and concerned local EC) and the local regulatory authorities, if requested. Violations (deviations) of the protocol that reveal systematic non-compliance or lead to critical patient safety issues require appropriate corrective and preventive action (CAPA) which will be overseen by project management.

10.7 Monitoring, auditing and inspecting

Monitoring is a quality control (QC) activity which involves a system of ongoing checks to detect failures, to correct them, and prevent reoccurrence of the failure. The overall goal of monitoring is to produce complete and consistent clinical data to safeguard patient safety, data integrity and patients´ rights. The monitoring activities ensure the prospectively defined level of performance of the targets identified for the study. Monitoring is being conducted by an appropriate mix of:

1. On-site monitoring visit: In person evaluation carried out by CRO personnel at the investigational site. On-site monitoring visits will focus on reviewing completeness and accuracy of Informed Consent Forms, source document verification (SDV) and Source Data Review (SDR) of original records, and other issues that may occur during the course of the clinical trial.

2. Off-site monitoring or remote monitoring: Evaluation carried out by CRO personnel outside the investigative site. It is a centralized review of individual site data. Data check is accomplished through regular phone monitoring with the study site personnel using monitoring questionnaires.

3. Central monitoring: Review of centralized data focusing on risk indicator data between investigative sites within a clinical trial or across studies. This review may be performed by medical monitors, data managers, project managers, statisticians.

The detailed monitoring processes are described in the study monitoring plan.

10.8 Premature closure of the study

This study may be prematurely terminated, if in the opinion of the investigator or the sponsor, there is sufficient cause. Written notification documenting the reason for study termination will be provided to the investigator or the sponsor by the terminating party.
Circumstances that may warrant termination include, but are not limited to:

- Failure to enter patients at an acceptable rate;
- Insufficient adherence to protocol requirements;
- Insufficient complete and/or evaluable data.

Should the study be closed prematurely, all study materials must be returned to Rna Diagnostics. The ethics committee and competent authorities (if required) will be notified by the CRO / SMOs according to the regulatory guidelines.
Data collection from patients recruited under previous BREVITY protocol versions (before the change of biopsy collection timepoints and re-start of BREVITY) will be stopped and further participation of these patients will be terminated. Sites that recruited patients under these previous protocol versions will be notified to implement close-out measures at the site. For these patients, only data collected up to surgery may be used (separately from the newly recruited patients after the change of the biopsy collection timepoints).

10.9 Data quality assurance

The CRO is responsible for the data management of this study including quality checking of the data. All participant data relating to the study will be recorded on electronic CRF.

The investigator is responsible for verifying that data entries are accurate and correct by electronically signing the CRF. The investigator must maintain accurate documentation (source data) that supports the information entered in the CRF as well as he/she must permit study-related monitoring, audits, IEC review, and regulatory agency inspections and provide direct access to source data documents. Source documents provide evidence for the existence of the participant and substantiate the integrity of the data collected. Source documents are filed at the investigator's site. Data reported on the eCRF that are transcribed from source documents must be consistent with the source documents or the discrepancies must be explained. The investigator may need to request previous medical records or transfer records, depending on the study. Also, current medical records must be available.

During onsite monitoring visits investigator(s) and their relevant staff must be available. The clinical monitors will perform an ongoing off-site combined with a risk-assessed on-site monitoring to confirm that data entered into the CRF by authorized site personnel are accurate, complete, and selected critical data are verifiable from source documents, that the safety and rights of participants are being protected; and that the study is being conducted in accordance with the currently approved protocol and any other study agreements, ICH-GCP, the standard "in-vitro diagnostic medical devices – clinical performance studies using specimens from human subject – Good study practice (ISO 209162019)", where applicable, and all applicable regulatory requirements.

10.10 Record retention

The investigator will maintain all study records according to ICH-GCP and the standard "in-vitro diagnostic medical devices – clinical performance studies using specimens from human subject – Good study practice (ISO 209162019)" where applicable. Records and documents, including signed ICFs, pertaining to the conduct of this study must be retained by the investigator for 10 years after study completion or discontinuation unless local regulations or institutional policies require a longer retention period. No records may be destroyed during the retention period without the written approval of the sponsor and no records may be transferred to another location or party without written notification to the sponsor.

The CRO/SMO and the sponsor will notify investigators if they are no longer required to maintain the files. If the investigator withdraws from the responsibility of keeping the study records, custody must be transferred to a person willing to accept the responsibility and this must be documented in writing to the sponsor.

10.11 Publication policy

Publications, papers, abstracts or presentations related to the study and the results (collectively, "Publications") will be prepared and submitted for publication jointly by the parties (participating investigators and Rna Diagnostics), or jointly by both parties in collaboration with other clinics performing the study internationally, subject to the following:

- No paper, abstract, presentation or any other document related to the study or the results will be submitted for publication without the express written consent of Rna Diagnostics, such consent to be at the sole and absolute discretion of Rna Diagnostics.
- All publications must be submitted by the authors to Rna Diagnostics for review and comment at least ninety (90) days prior to submission to any publication or third party disseminator.
- Rna Diagnostics may by written notification require the authors to postpone the publication of the proposed publication in order for Rna Diagnostics to take the steps necessary to protect its intellectual property rights. Upon receipt of such written notice, the authors shall delay the publication or presentation of the proposed publication for the period of time specified in

the notice, provided that such period shall not exceed ninety (90) days from the date the written notice is provided by Rna Diagnostics.

- Rna Diagnostics shall have the right to require amendments to any such proposed publication: (i) to ensure that proprietary information is not inadvertently divulged; or (ii) to enable intellectual property rights to be secured; or (iii) to enable relevant supplementary information to be provided by Rna Diagnostics. The authors shall be required to amend or delete any statement in a proposed publication pursuant to (i) or (ii) above, and to add any such relevant supplementary information pursuant to (iii) above.
- The CRO / SMOs and the participating centres also undertake, and shall ensure that their employees, personnel and advisors and investigators involved in the services fulfil all obligations set out in this publication policy.

The CRO and sponsor warrant that, in accordance with requirements of the International Committee of Medical Journal Editors (ICMJE) for publication of study results, will register the study in www.clinicaltrials.gov prior to the enrolment of the first patient.

10.12 Indemnity

Sufficient insurance will be provided by Rna Diagnostics Inc. The CRO/SMOs will forward to the Ethics Committees and every investigator a copy of the insurance policy covering its and any other participating party’s liabilities.

10.13 Patient expense reimbursement

Expenses such as transportation and parking fees on days when RDA biopsies are scheduled will be reimbursed to the participating patient.

10.14 Data privacy and protection

The BREVITY trial will be conducted in compliance with the Personal Information Protection and Electronic Data Act (PIPEDA) and the Health Insurance Portability and Accountability Act (HIPAA) respectively for Canada and the US.

11. APPENDICES

APPENDIX A: REFERENCES

[1] Parissenti AM, Guo B, Pritzker LB, Pritzker KP, Wang X, Zhu M, Shepherd LE, Trudeau ME: Tumor RNA disruption predicts survival benefit from breast cancer chemotherapy. Breast cancer research and treatment 2015, 153:135-44.
[2] Narendrula R, Mispel-Beyer K, Guo B, Parissenti AM, Pritzker LB, Pritzker K, Masilamani T, Wang X, Lanner C: RNA disruption is associated with response to multiple classes of chemotherapy drugs in tumor cell lines. BMC cancer 2016, 16:146.
[3] Boileau JF: RNA Disruption in Fine Needle Aspirate Tumour Samples Obtained During Chemotherapy. In Preparation.
[4] Pritzker K, Pritzker L, Generali D, Bottini A, Cappelletti MR, Guo B, Parissenti A, Trudeau M: RNA Disruption and Drug Response in Breast Cancer Primary Systemic Therapy. Journal of the National Cancer Institute Monographs 2015, 2015:76-80.
[5] Gebhart G, Gamez C, Holmes E, Robles J, Garcia C, Cortes M, de Azambuja E, Fauria K, Van Dooren V, Aktan G, Coccia-Portugal MA, Kim SB, Vuylsteke P, Cure H, Eidtmann H, Baselga J, Piccart M, Flamen P, Di Cosimo S: 18F-FDG PET/CT for early prediction of response to neoadjuvant lapatinib, trastuzumab, and their combination in HER2-positive breast cancer: results from Neo-ALTTO. Journal of nuclear medicine : official publication, Society of Nuclear Medicine 2013, 54:1862-8.
[6] Connolly RM, Leal JP, Goetz MP, Zhang Z, Zhou XC, Jacobs LK, Mhlanga J, O JH, Carpenter J, Storniolo AM, Watkins S, Fetting JH, Miller RS, Sideras K, Jeter SC, Walsh B, Powers P, Zorzi J, Boughey JC, Davidson NE, Carey LA, Wolff AC, Khouri N, Gabrielson E, Wahl RL, Stearns V: TBCRC 008: early change in 18F-FDG uptake on PET predicts response to preoperative systemic therapy in human epidermal growth factor receptor 2-negative primary operable breast cancer. Journal of nuclear medicine : official publication, Society of Nuclear Medicine 2015, 56:31-7.
[7] Groheux D, Mankoff D, Espie M, Hindie E: (1)(8)F-FDG PET/CT in the early prediction of pathological response in aggressive subtypes of breast cancer: review of the literature and recommendations for use in clinical trials. European journal of nuclear medicine and molecular imaging 2016, 43:983-93.
[8] von Minckwitz G, Blohmer JU, Costa SD, Denkert C, Eidtmann H, Eiermann W, Gerber B, Hanusch C, Hilfrich J, Huober J, Jackisch C, Kaufmann M, Kummel S, Paepke S, Schneeweiss A, Untch M, Zahm DM, Mehta K, Loibl S: Response-guided neoadjuvant chemotherapy for breast cancer. Journal of clinical oncology : official journal of the American Society of Clinical Oncology 2013, 31:3623-30.
[9] Miquel-Cases A, Retel VP, Lederer B, von Minckwitz G, Steuten LM, van Harten WH: Exploratory Cost-Effectiveness Analysis of Response-Guided Neoadjuvant Chemotherapy for Hormone Positive Breast Cancer Patients. PloS one 2016, 11:e0154386.
[10] Cortazar P, Zhang L, Untch M, Mehta K, Costantino JP, Wolmark N, Bonnefoi H, Cameron D, Gianni L, Valagussa P, Swain SM, Prowell T, Loibl S, Wickerham DL, Bogaerts J, Baselga J, Perou C, Blumenthal G, Blohmer J, Mamounas EP, Bergh J, Semiglazov V, Justice R, Eidtmann H, Paik S, Piccart M, Sridhara R, Fasching PA, Slaets L, Tang S, Gerber B, Geyer CE, Jr., Pazdur R, Ditsch N, Rastogi P, Eiermann W, von Minckwitz G: Pathological complete response and long-term clinical benefit in breast cancer: the CTNeoBC pooled analysis. Lancet 2014.
[11] Fitzal F, Sporn EP, Draxler W, Mittlbock M, Taucher S, Rudas M, Riedl O, Helbich TH, Jakesz R, Gnant M: Preoperative core needle biopsy does not increase local recurrence rate in breast cancer patients. Breast Cancer Res Treat 2006, 97:9-15.

[12] Knight R, Horiuchi K, Parker SH, Ratzer ER, Fenoglio ME: Risk of needle-track seeding after diagnostic image-guided core needle biopsy in breast cancer. JSLS 2002, 6:207-9.
[13] Rna Diagnostics: Risk Assessment and Risk Management of the RNA Disruption Assay (RDA) - Document Number RDx_RMT_01.
[14] Rna Diagnostics: Statistical Analysis Plan (BREVITY Trial).
[15] Chow S-C, Shao J, Wang H: Sample size calculations in clinical research. 2nd ed. Boca Raton: Chapman & Hall/CRC, 2008.
[16] Newcombe RG: Two-sided confidence intervals for the single proportion: comparison of seven methods. Statistics in medicine 1998, 17:857-72.
[17] Fleiss JL, Levin B, Paik MC: Statistical methods for rates and proportions. 3rd ed. Hoboken, N.J.: J. Wiley, 2003.
[18] Klein JP, and Moeschberger M. L. : Survival analysis: statistical methods for censored and truncated data. Springer, New York, 2003.
[19] Dmitrienko A, Tamhane AC, Bretz F: Multiple testing problems in pharmaceutical statistics. Boca Raton, FL: Chapman & Hall/CRC, 2010.
[20] Rubin DB, and Schenker, N. : Multiple imputation for interval estimation from simple random samples with ignorable nonresponse. Journal of the American Statistical Association 81394 (1986): 366-374.
[21] National Research Council (U.S.). Panel on Handling Missing Data in Clinical Trials., National Research Council (U.S.). Committee on National Statistics., National Academies Press (U.S.): The prevention and treatment of missing data in clinical trials. Washington, D.C.: National Academies Press, 2010.

APPENDIX B: SAMPLE COLLECTION TIME POINTS, PROCESSING, STORAGE AND TRANSPORT

Table 1. Biopsy Collection Time Points		
Biopsy	*1st core needle biopsy for RDA (2 specimens)*	*2nd core needle biopsy for RDA (2 specimens)*
Biopsy timing by type of drug schedule	*35 days +/- 4 days*	***If no change of drugs:***<br>*55 days +/- 5 days*<br><br>***If there is a change of drugs:***<br>*2 to 3 weeks after start of new drugs as follows depending on drug regimen:*<br>• *3-weekly: at 16 days +/- 2 days*<br>• *Bi-weekly: at day of 2nd dose preferably before drug admin.*<br>• *Weekly: at day of 4th dose preferably before drug admin.*

SPECIMEN LABELLING, COLLECTION, HANDLING AND TRANSPORTATION GUIDELINES

Specimen Labelling

Rna Diagnostics will provide the biopsy specimen collection kits. Use one kit per patient at each collection point. Each kit will consist of one Cryo Transport box containing two (2), 2mL Sarstedt screw-cap tubes labelled A and B, filled with 1 mL of tumour biopsy fixative (RNAlater® Tissue Collection Solution) each. All kits will be sent with labels. Labels for the tubes will be pre-printed with (at a minimum) identifiers for clinical site ID, and the lot number, and the expiry date of fixative used. **Record the four digits of Subject ID and date that the sample will be taken (DD/MM/YY) using an indelible black marker**. Attach the labels to the sides of the Sarstedt screw-cap tubes at the time of obtaining the core biopsy.

Specimen Collection

Perform the procedure aseptically and wear gloves during handling of all materials (to prevent RNase contamination). Take 2 core biopsy specimens (from the same tumour). After collection, transfer each core biopsy immediately into a separate Sarstedt screw-cap tube containing the core biopsy fixative. After collection, tightly seal the specimen containers. Invert the 2 tubes 3 times to mix the biopsy material in the fixative. Submit **all specimens for testing with a completed RDA requisition form.**

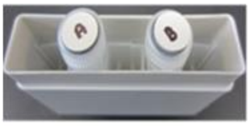

Labelled Sarstedt Screw-Cap Tubes containing RNAlater Tissue Collection Solution

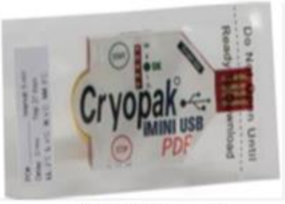

iMINI USB pdf temperature logger

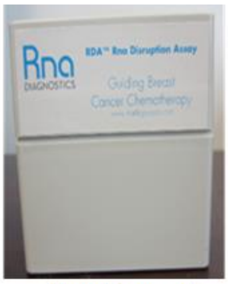

Cryo Transport Box

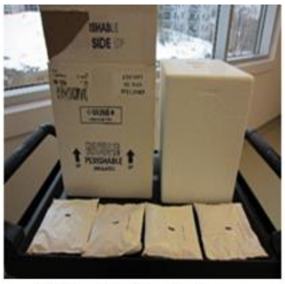

Shipping Container and Cold packs

<u>Specimen Handling</u>
Once specimens are collected in fixative (RNAlater® Tissue Collection Solution), **place them at room temperature overnight in the Cryo transport box** and **then store the specimens at – 10 °C or less until they are shipped**.

<u>Specimen Packing</u>
Ship the specimens to the Rna Diagnostics laboratory in Sudbury, Ontario, Canada preferably on a Monday. Specimens from multiple patients can be shipped in one shipment on the day of collective shipment, which will be either when about 10 patients have been accrued in the same centre, or at day 90 (biopsies should not be kept in the freezer for longer than 90 days). The SMOs/CROs will notify the centres of a due shipment on a quarterly basis.
Please refer to and read carefully the "Biopsy Specimens Packing Instructions" document attached to the protocol for a detailed description of the steps to follow for biopsy packing on the shipping date.

<u>Specimen Transportation/Shipping Guidelines</u>
For shipments from US, fill out the FedEx International Way bill and the commercial invoice (CI). Attach a copy of the Power of Attorney (POA) document confirming FedEx to act as broker for Rna Diagnostics Inc. Place the FedEx waybill, three copies of the CI, POA confirmation document, Safety Data Sheet (SDS) of RNA*later* on the outside of the cardboard box for customs clearance.
For shipments within Canada, fill out the Intra-Canada Waybill.
Email the shipment tracking number to rda@rnadiagnostics.com in order to notify Rna Diagnostics of the shipment of the core biopsy specimens.
<u>Shipments from US,</u> fill out the FedEx International Waybill according to the following instructions:

- (1) Enter all necessary "SENDER" information (**date, name, company/institution and address**)
- (2) Enter "RECIPIENT" information as follows:

- **Rna Diagnostics**
  56 Walford Rd
  2nd Floor North
  Sudbury ON
  Canada
  P3E 2H3

Telephone: (705) 523 7300 Ext 1144 or 1182

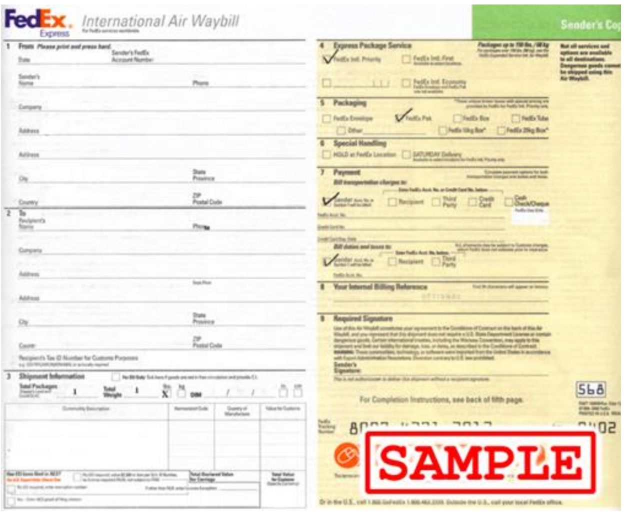

FedEx International Waybill

- (3) Identify the **total number of packages, weight and dimensions of shipped goods** along with the **total value of goods** for customs purposes
- (3) In the commodity description, identify the contents/biopsies as **Human Tissue biopsy "Exempt Human Specimens" used for diagnostic purposes (non-infectious)** **(as identified by exception 3.6.2.2.3.8 in IATA DGR Hazard class 6, Division 2, 61st edition, January 2020)**
- (4) In the Express Freight Service Section, choose **FedEx International Priority**
- (5) In the Packaging section, choose **Other** and enter **Box**
- (6a) When asked if this shipment contains dangerous goods, check **No**
- (6b) **Do not complete broker selection fields**; FedEx will act as the broker for FedEx International Priority
- (7a) Bill transportation charges to **Recipient**, and indicate the following FedEx Account Number:
  - **FedEx Account #: 32916 7593**
- (7b) Bill duties and taxes to **Recipient**, and indicate the following FedEx Account Number:
  - **FedEx Account #: 32916 7593**
- (8) Sign where prompted for the Sender's signature

Fill out the commercial invoice (CI) according to the following instructions:

- Enter **FedEx way bill number** in "International Airwaybill Number"
- Enter the **date of shipping** in "Date of Exportation"

- Enter **sender information** (Name, street, city, state, ZIP/Postal code, country, phone number) jn “Shipper/Exporter”
- Enter the following in “Consignee” or “Recipient”

  Rna Diagnostics
  56 Walford Road
  2nd Floor North,
  Sudbury, ON, P3E 2H3
  Canada
  Phone: 705 523 7300 Ext 1144 or 1182

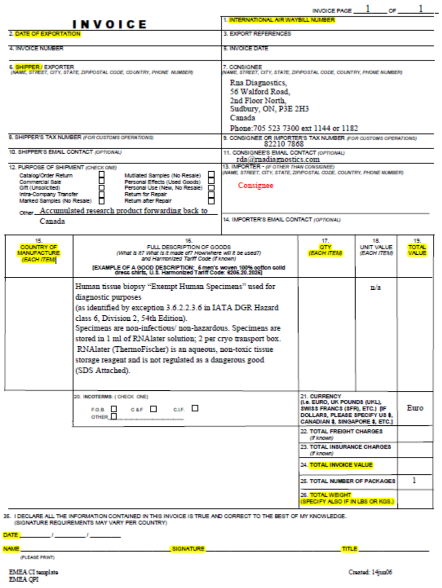

INVOICE PAGE 1 OF 1

INVOICE

2. DATE OF EXPORTATION	1. INTERNATIONAL AIR WAYBILL NUMBER
	3. EXPORT REFERENCES
4. INVOICE NUMBER	5. INVOICE DATE
6. SHIPPER / EXPORTER (NAME, STREET, CITY, STATE, ZIP/POSTAL CODE, COUNTRY, PHONE NUMBER)	7. CONSIGNEE (NAME, STREET, CITY, STATE, ZIP/POSTAL CODE, COUNTRY, PHONE NUMBER)<br>Rna Diagnostics,<br>56 Walford Road,<br>2nd Floor North,<br>Sudbury, ON, P3E 2H3<br>Canada<br>Phone:705 523 7300 ext 1144 or 1182
8. SHIPPER'S TAX NUMBER (FOR CUSTOMS OPERATIONS)	9. CONSIGNEE OR IMPORTER'S TAX NUMBER (FOR CUSTOMS OPERATIONS)<br>82210 7868
10. SHIPPER'S EMAIL CONTACT (OPTIONAL)	11. CONSIGNEE'S EMAIL CONTACT (OPTIONAL)<br>rda@rnadiagnostics.com
12. PURPOSE OF SHIPMENT (CHECK ONE)<br>Catalog/Order Return ☐ Mutilated Samples (No Resale) ☐<br>Commercial Sale ☐ Personal Effects (Used Goods) ☐<br>Gift (Unsolicited) ☐ Personal Use (New, No Resale) ☐<br>Intra-Company Transfer ☐ Return for Repair ☐<br>Marked Samples (No Resale) ☐ Return after Repair ☐<br>Other Accumulated research product forwarding back to Canada	13. IMPORTER - (IF OTHER THAN CONSIGNEE) (NAME, STREET, CITY, STATE, ZIP/POSTAL CODE, COUNTRY, PHONE NUMBER)<br>Consignee
	14. IMPORTER'S EMAIL CONTACT (OPTIONAL)

15. COUNTRY OF MANUFACTURE (EACH ITEM)	16. FULL DESCRIPTION OF GOODS (What is it? What is it made of? How/where will it be used?) and Harmonized Tariff Code (if known) [EXAMPLE OF A GOOD DESCRIPTION: 6 men's woven 100% cotton solid dress shirts, U.S. Harmonized Tariff Code: 6205.20.2026]	17. QTY (EACH ITEM)	18. UNIT VALUE (EACH ITEM)	19. TOTAL VALUE
	Human tissue biopsy “Exempt Human Specimens” used for diagnostic purposes (as identified by exception 3.6.2.2.3.6 in IATA DGR Hazard class 6, Division 2, 54th Edition). Specimens are non-infectious/ non-hazardous. Specimens are stored in 1 ml of RNAlater solution; 2 per cryo transport box. RNAlater (ThermoFischer) is an aqueous, non-toxic tissue storage reagent and is not regulated as a dangerous good (SDS Attached).		n/a	
	20. INCOTERMS: (CHECK ONE) F.O.B. ☐ C&F ☐ C.I.F. ☐ OTHER ☐	21. CURRENCY (i.e. EURO, UK POUNDS (UKL), SWISS FRANCS (SFR), ETC.) [IF DOLLARS, PLEASE SPECIFY US $, CANADIAN $, SINGAPORE $, ETC.]		Euro
		22. TOTAL FREIGHT CHARGES (if known)		
		23. TOTAL INSURANCE CHARGES (if known)		
		24. TOTAL INVOICE VALUE		
		25. TOTAL NUMBER OF PACKAGES		1
		26. TOTAL WEIGHT (SPECIFY ALSO IF IN LBS OR KGS.)		

35. I DECLARE ALL THE INFORMATION CONTAINED IN THIS INVOICE IS TRUE AND CORRECT TO THE BEST OF MY KNOWLEDGE. (SIGNATURE REQUIREMENTS MAY VARY PER COUNTRY)

DATE ____ / ____ / ____

NAME ________ SIGNATURE ________ TITLE ________
(PLEASE PRINT)

EMEA CI template
EMEA QPI
Created: 14jun06

- Enter “**82210 7868**” in “Consignee or importer’s Tax Number (for customs operations)
- Enter rda@rnadiagnostics.com in “Consignee’s Email Contact”
- Purpose of Shipment, under OTHER write **“Accumulated research product forwarding back to Canada”**
- Enter “**Consignee**” in the Importer section
- In “Country of Manufacture” write the country the shipment is sent from
- Enter the following in the “Full description of Goods”:

Human tissue biopsy “Exempt Human Specimens” used for diagnostics purposes (as identified by exception 3.6.2.2.3.8 in IATA DGR Hazard Class 6, Division 2, 61st Edition, January 2020). Specimens are non-infectious/ non-hazardous. Specimens are stored in 1ml RNAlater solution; 2 per cryo transport box. RNAlater (Thermo Fischer) is an aqueous, non-toxic tissue storage reagent and is not regulated as a dangerous good (SDS attached).

- Enter the total number of cryo transport boxes in “Qty”
- Enter **n/a** in “Unit Value”
- Enter the value in “Total Value”
- Enter the currency used in “Currency”
- Enter the total value in “Total invoice value”
- Enter **1** in the “Total Number of Packages”
- Enter the weight of the package and specify units (lbs or kgs) in “Total Weight”
- Complete the declaration section with the date, name, signature and title of the sender.
- Place 3 copies of the CI, the SDS of RNA*later*, the FedEx “Power of Attorney” statement, the packing list and the completed FedEx waybill in a waterproof pack on the outside of the cardboard portion of the shipment box.

All international shipments must adhere to the International Air Transport Association Dangerous Goods Regulations (IATA DGR) and the Canadian Transportation of Dangerous Goods Regulations (TDGR) guidelines.

<u>For shipments within Canada</u>, fill out the FedEx Intra Waybill according to the following instructions:

- (1) Enter all necessary “SENDER” information (**date, name, company/institution and address**)
- (2) Enter “RECIPIENT” information as follows:

- Rna Diagnostics
  56 Walford Rd
  2nd Floor North
  Sudbury ON
  Canada
  P3E 2H3

Telephone: (705) 523 7300 Ext 1144 or 1182

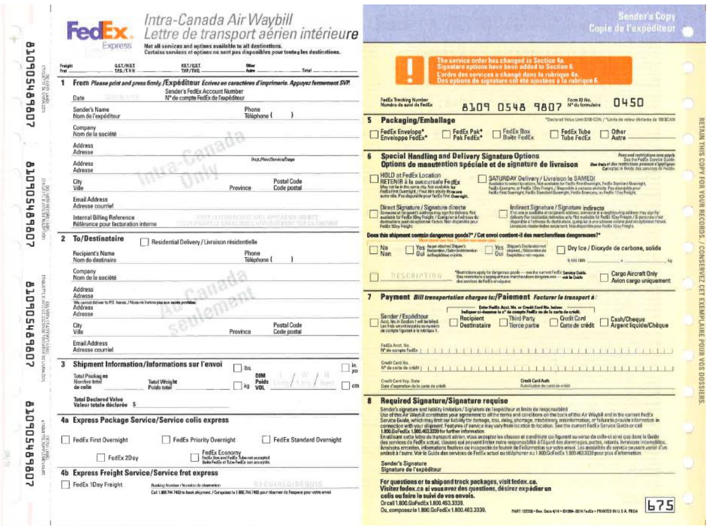

- (3) Identify the **total number of packages, weight (lbs or kg) and dimensions of shipped goods (in or cm)** along with the **total declared value of goods**
- (4a) In the Express Package Service Section, choose **FedEx Priority Overnight**
- (5) In the Packaging section, choose **Other** and enter **Box**
- (6) When asked if this shipment contains dangerous goods, check **No**
- (7) Bill transportation charges to **Recipient**, and indicate the following FedEx Account Number:
  - **FedEx Account #: 32916 7593**
- (8) Sign where prompted for the Sender's signature

APPENDIX C: INVESTIGATOR AGREEMENT (mandatory)

INVESTIGATOR AGREEMENT

This constitutes a binding agreement between the sponsor and investigator and must be completed by each investigator before a study is opened at a participating site.

I have read the Protocol:

**Study Title:**	RNA Disruption Assay (RDA) - **Br**east cancer **R**esponse **EV**aluation for **I**ndividualized **T**herap**Y** (*BREVITY*)
**Version/ date:**	Version 18 (USA) – March 2022

I agree to conduct the study as detailed herein and in compliance with ICH-GCP, the standard "in-vitro diagnostic medical devices – clinical performance studies using specimens from human subjects – Good study practice (ISO 209162019)" and applicable ethical requirements and to ensure that all persons assisting with the study are adequately informed about the protocol and their study-related duties and functions.

____
Investigator (printed name)

______________________________________ ____________________________
Investigator signature Date dd-MON-yyyy

____
Investigational site or name of institution and location (printed)

Supplementary Table 1: BREVITY Training Set Data Table

Phase I Patient	Hormone Receptor Status	HER2 Receptor Status	ypT	ypN	Pathological Complete Response (pCR)	RDI Score A	RDI Score B	RDI Score C	RDI Score D	Maximum RDI Score	Tumor Stage	Grade	Histology	Menopausal status
1	negative	negative	ypT1	ypN1a	no	1.1	1.0	1.8	8.9	8.9	IIB	No info	Infiltrating ductal	premenopausal
2	positive	positive	ypT0	ypN0	yes (ypT0-ypN0)	n/a	22.0	8.1	4.4	22	IIB	No info	Infiltrating ductal	premenopausal
3	positive	positive	ypT1	ypN0	no	16.7	4.7	4.4	9.0	16.7	IIIA	Grade 2	Infiltrating ductal	postmenopausal
4	negative	negative	ypT2	ypN0	no	0.9	0.9	1.1	0.9	1.1	IIA	Grade 3	Infiltrating ductal	postmenopausal
5	negative	negative	ypT2	ypN0	no	1.7	1.6	1.4	1.1	1.7	IIA	Grade 2	Infiltrating ductal	postmenopausal
6	negative	negative	ypT2	ypN2a	no	1.2	0.9	1.6	1.0	1.6	IIIA	Grade 3	Infiltrating ductal	premenopausal
7	negative	positive	ypTis	ypN0	no	3.1	1.2	8.5	6.1	8.5	IIA	Grade 2	Infiltrating ductal	postmenopausal
8	negative	negative	ypT0	ypN0	yes (ypT0-ypN0)	3.0	2.0	10.0	9.1	10.0	IIB	Grade 3	Infiltrating ductal	postmenopausal
9	positive	positive	ypT1	ypN1mi	no	4.6	2.7	2.9	3.2	4.6	IIIA	Grade 2	Infiltrating ductal	perimenopausal
10	positive	positive	ypTis	ypN0	no	5.4	4.7	10.7	n/a	10.7	IIA	Grade 3	Infiltrating ductal	postmenopausal
11	negative	positive	ypT1	ypN1mi	no	2.5	2.7	9.7	5.3	9.7	IIIB	Grade 3	Other: inflammatory	premenopausal
12	positive	negative	ypT3	ypN3a	no	2.3	1.5	3.0	1.5	3.0	IIB	Grade 2	Infiltrating ductal	postmenopausal
13	positive	positive	ypT1	ypN0	no	2.2	10.1	6.3	7.7	10.1	IIIA	Grade 3	Infiltrating ductal	premenopausal
14	negative	positive	ypT1	ypN0	no	5.4	7.0	3.5	5.0	7.0	IIA	Grade 3	Infiltrating ductal	postmenopausal
15	positive	negative	ypT0	ypN0	yes (ypT0-ypN0)	1.1	4.3	2.5	4.6	4.6	IIIA	Grade 3	Infiltrating ductal	premenopausal
16	positive	negative	ypT3	ypN1a	no	5.2	4.9	3.1	3.0	5.2	IIIA	Grade 2	Infiltrating lobular	postmenopausal
17	negative	negative	ypT1	ypN0	no	1.9	2.1	1.5	1.3	2.1	IIA	Grade 3	Infiltrating ductal	premenopausal
18	negative	negative	ypT2	ypN1a	no	4.8	3.9	8.7	2.7	8.7	I	Grade 3	Infiltrating ductal	postmenopausal
19	negative	negative	ypT2	ypN1a	no	n/a	5.4	2.4	3.1	5.4	IIIA	Grade 3	Infiltrating ductal	premenopausal
20	negative	negative	ypT0	ypN0	yes (ypT0-ypN0)	3.5	16.4	8.2	10.0	16.4	IIA	Grade 2	Infiltrating ductal	premenopausal
21	negative	positive	ypT0	ypN0	yes (ypT0-ypN0)	7.5	1.8	6.5	17.8	17.8	IIA	Grade 3	Other: invasive not other specified	premenopausal

22	positive	positive	ypT0	ypN0	yes (ypT0-ypN0)	2.9	1.0	7.5	4.3	7.5	No info	Grade 3	Infiltrating lobular	postmenopausal
23	negative	negative	ypT0	ypN0	yes (ypT0-ypN0)	1.0	2.2	5.1	4.0	5.1	IIA	Grade 3	Infiltrating ductal	premenopausal
24	negative	negative	ypT0	ypN0	yes (ypT0-ypN0)	4.3	8.5	10.8	n/a	10.8	IIA	Grade 2	Infiltrating lobular	postmenopausal
25	negative	positive	ypTis	ypN0	no	1.6	2.1	4.2	12.7	12.7	No info	Grade 2	Infiltrating ductal	premenopausal
26	positive	negative	ypT3	ypN0	no	4.8	3.7	5.0	10	10.0	No info	Grade 3	Infiltrating ductal	postmenopausal
27	negative	negative	ypT0	ypN0	yes (ypT0-ypN0)	0.9	0.7	38.9	9.4	38.9	No info	Grade 2	Infiltrating ductal	postmenopausal
28	negative	positive	ypT0	ypN0	yes (ypT0-ypN0)	6	4.2	7.8	3.9	7.8	IIA	Grade 2	Infiltrating ductal	postmenopausal
29	positive	negative	ypT1	ypN1a	no	1.7	1.7	n/a	1.8	1.8	IIA	Grade 2	Infiltrating ductal	premenopausal
30	positive	negative	ypT2	ypN0	no	9.1	8.7	4.9	8.1	9.1	IIA	Grade 2	Infiltrating ductal	postmenopausal
31	positive	negative	ypT2	ypN0	no	0.5	0.8	0.7	1.5	1.5	IIA	Grade 3	Infiltrating ductal	premenopausal
32	negative	negative	ypT0	ypN0	yes (ypT0-ypN0)	6.3	6.4	5.8	4.8	6.4	IIA	Grade 3	Infiltrating ductal	perimenopausal
33	negative	negative	ypT0	ypN0	yes (ypT0-ypN0)	n/a	16.0	5.2	5.8	16.0	IIA	Grade 2	Infiltrating ductal	premenopausal
34	positive	positive	ypT0	ypN0	yes (ypT0-ypN0)	1.4	1.2	22.6	7.7	22.6	I	Grade 2	Infiltrating ductal	premenopausal
35	positive	positive	ypT0	ypN0	yes (ypT0-ypN0)	1.6	1.3	n/a	5.1	5.1	IIIB	Grade 2	Infiltrating ductal	postmenopausal
36	positive	positive	ypT2	ypN2a	no	5.1	3.9	2.8	5.4	5.4	IIIA	Grade 2	Infiltrating ductal	postmenopausal
37	positive	negative	ypT0	ypN0	yes (ypT0-ypN0)	5.1	5.1	2.4	4.9	5.1	I	Grade 3	Infiltrating ductal	premenopausal
38	negative	positive	ypT0	ypN0	yes (ypT0-ypN0)	9.0	n/a	n/a	5.2	9.0	IIA	Grade 3	Infiltrating ductal	premenopausal
39	negative	negative	ypT1	ypN1a	no	0.7	0.7	0.9	0.9	0.9	IIA	Grade 3	Infiltrating ductal	premenopausal
40	negative	negative	ypT1	ypN0	no	1.4	2.0	1.3	3.8	3.8	IIA	Grade 3	Other: poorly differentiated breast cancer	premenopausal
41	negative	negative	ypT1	ypN0	no	1.2	1.0	2.4	4.5	4.5	I	Grade 3	Other: invasive	postmenopausal
42	negative	negative	ypT1	ypN0	no	1.9	1.5	13.3	n/a	13.3	I	Grade 3	Infiltrating ductal	postmenopausal
43	positive	positive	ypT3	ypN2a	no	3.2	2.5	0.9	16.7	16.7	IIA	Grade 2	Infiltrating lobular	unknown
44	negative	negative	ypTis	ypN0	no	1.7	7.4	4.6	8.4	8.4	IIA	Grade 3	Infiltrating ductal	postmenopausal

45	negative	positive	ypT0	ypN0	yes (ypT0-ypN0)	1.6	4.1	1.6	2.9	4.1	IIB	Grade 3	Infiltrating ductal	postmenopausal
46	negative	negative	ypT0	ypN0	yes (ypT0-ypN0)	5.3	2.0	4.5	n/a	5.3	IIB	Grade 3	Infiltrating ductal	postmenopausal
47	negative	negative	ypT0	ypN0	yes (ypT0-ypN0)	0.8	2.2	3.5	3.8	3.8	IIB	Grade 3	Other: not specific	postmenopausal
48	negative	negative	ypT1	ypN0	no	1.0	2.1	4.0	3.7	4.0	IIA	Grade 3	Infiltrating ductal	postmenopausal
49	positive	negative	ypT1	ypN1a	no	0.9	1.0	0.9	0.9	1.0	IIB	Grade 3	Infiltrating ductal	unknown
50	negative	negative	ypT1	ypN0	no	1.1	2.3	1.9	3.2	3.2	IIB	Grade 2	Infiltrating ductal	postmenopausal
51	positive	positive	ypT1	ypN0	no	0.9	0.8	3.5	6.4	6.4	I	Grade 2	Infiltrating ductal	premenopausal
52	positive	positive	ypT1	ypN0	no	3.3	9.9	11.2	6.4	11.2	IIA	Grade 2	Infiltrating ductal	postmenopausal
53	positive	negative	ypTis	ypN0	no	2.8	2.8	4.9	5.6	5.6	IIA	Grade 2	Infiltrating ductal	premenopausal
54	positive	negative	ypT1	ypN1a	no	3.1	3.9	4.5	4.1	4.5	IIA	Grade 2	Infiltrating ductal	premenopausal
55	positive	negative	ypT1	ypN1a	no	1.6	2.7	4.5	5.5	5.5	IIB	Grade 2	Infiltrating ductal	premenopausal
56	negative	negative	ypT0	ypN0	yes (ypT0-ypN0)	4.6	5.0	6.8	3.3	6.8	I	Grade 3	Infiltrating ductal	postmenopausal
57	negative	negative	ypT2	ypN0	no	4.8	3.2	4.6	2	4.8	IIA	Grade 2	Infiltrating ductal	postmenopausal
58	positive	negative	ypT1	ypN2a	no	3.4	9.7	4.7	12.9	12.9	IIIA	Grade 2	Infiltrating ductal	postmenopausal
59	negative	positive	ypTis	ypN0	no	5.4	5.5	1.7	2.2	5.5	IIB	Grade 3	Infiltrating ductal	postmenopausal
60	positive	negative	ypT2	ypN1a	no	5.2	3.7	2.7	8.3	8.3	IIIB	Grade 2	Infiltrating ductal	premenopausal
61	positive	positive	ypT0	ypN0	yes (ypT0-ypN0)	3.5	4.3	6.7	18.8	18.8	IIIA	Grade 2	Infiltrating ductal	postmenopausal
62	negative	negative	ypT2	ypN1a	no	1.2	6.2	1.8	1.5	6.2	IIA	Grade 3	Infiltrating ductal	premenopausal
63	negative	positive	ypT0	ypN0	yes (ypT0-ypN0)	6.0	9.1	10.7	n/a	10.7	IIA	Grade 3	Infiltrating ductal	postmenopausal
64	negative	negative	ypT0	ypN0	yes (ypT0-ypN0)	1.3	1.2	n/a	1.7	1.7	I	Grade 3	Infiltrating ductal	premenopausal
65	negative	positive	ypT0	ypN0	yes (ypT0-ypN0)	1.7	9.0	1.8	5.1	9.0	IIIA	Grade 2	Infiltrating ductal	postmenopausal
66	negative	positive	ypT1	ypN0	no	1.9	3.7	3.6	3	3.7	IIA	Grade 2	Infiltrating ductal	premenopausal
67	positive	positive	ypT1	ypN0	no	5.6	4.0	8.1	16.8	16.8	IIB	Grade 3	Infiltrating ductal	premenopausal
68	positive	positive	ypT0	ypN0	yes (ypT0-ypN0)	1.8	3.6	8.6	10.8	10.8	IIA	Grade 2	Infiltrating ductal	premenopausal

69	negative	negative	ypT1	ypN1a	no	0.9	1.7	0.9	1.3	1.7	IIIA	Grade 3	Other: infiltrating not specified	premenopausal
70	positive	positive	ypT2	ypN1mi	no	1.5	1.0	5.4	2.6	5.4	IIA	Grade 2	Other: infiltrating	postmenopausal
71	negative	negative	ypT1	ypN1a	no	1.2	1.1	4.1	6.2	6.2	IIB	No info	Infiltrating ductal	premenopausal
72	negative	positive	ypTis	ypN0	no	2.8	3.4	10.0	8.5	10.0	IIB	Grade 2	Infiltrating ductal	postmenopausal
73	negative	positive	ypT0	ypN0	yes (ypT0-ypN0)	10.7	4.2	4.9	3.7	10.7	IIB	Grade 2	Infiltrating ductal	premenopausal
74	negative	positive	ypTis	ypN2a	no	1.7	1.4	8.2		8.2	I	Grade 3	Infiltrating ductal	postmenopausal
75	negative	negative	ypT3	ypN0	no	0.9	n/a	1.0	1.0	1.0	IIA	Grade 3	Infiltrating ductal	postmenopausal
76	positive	negative	ypT1	ypN0	no	0.7	2.1	1.5	1.8	2.1	IIB	Grade 3		postmenopausal
77	positive	negative	ypT1	ypN0	no	3.9	2.8	5.7	14.7	14.7	IIA	Grade 3	Infiltrating ductal	postmenopausal
78	negative	positive	ypT1	ypN0	no	1.6	7.8	1.9	1.8	7.8	IIIA	Grade 3	Infiltrating ductal	postmenopausal
79	negative	negative	ypT1	ypN0	no	1.7	4.7	10.1	0.8	10.1	I	Grade 2	Other: invasive mammary carcinoma	postmenopausal
80	negative	negative	ypT0	ypN0	yes (ypT0-ypN0)	1.8	5.7	19	5.9	19.0	IIA	Grade 3	Infiltrating ductal	postmenopausal
